# Small within the largest: brain size and anatomy of the extinct *Neoepiblema acreensis*, a giant rodent from the Neotropics

**DOI:** 10.1098/rsbl.2019.0914

**Published:** 2020-02-12

**Authors:** José D. Ferreira, Francisco R. Negri, Marcelo R. Sánchez-Villagra, Leonardo Kerber

**Affiliations:** 1 Programa de Pós-Graduação em Biodiversidade Animal, Universidade Federal de Santa Maria, Santa Maria, Brazil; 2 Laboratório de Paleontologia, Campus Floresta, Universidade Federal do Acre, Cruzeiro do Sul, Acre, Brazil; 3 Palaeontological Institute and Museum, University of Zurich, Zurich, Switzerland; 4 Centro de Apoio à Pesquisa Paleontológica da Quarta Colônia (CAPPA), Universidade Federal de Santa Maria, São João do Polêsine, Brazil

**Keywords:** endocranium, Caviomorpha, palaeobiology, allometry, endocast

## Abstract

The ecomorphological diversity of caviomorph rodents in South America included giant forms, such as the chinchilloid *Neoepiblema acreensis* from the Upper Miocene of Brazil. The evolution of the brain anatomy and size of these animals can be now studied with non-invasive imaging techniques and exceptional fossils. Caviomorphs show diversity in the traits of the olfactory bulbs, cerebrum, cerebellum, cranial nerves, and blood vessels. *Neoepiblema acreensis* had a gyrencephalic brain, with an expansion of the frontal lobe, lacking an evident paraflocculus. Compared to the predictions based on extant taxa, even when considering taphonomical effects, *N. acreensis*, a rodent that weighted almost 80 kg, had a very low encephalization quotient compared to other rodents. The adaptive value of a low energetic cost and other ecological factors could explain the presence of a small brain in this giant rodent––a pattern we also hypothesize for other Neogene giant rodents.

## 1. Introduction

Caviomorpha is a diverse group of rodents from South America [1–3], derived from African forms that reached the continent during the Eocene [3,4]. During their long geographical isolation, caviomorphs evolved a broad ecological and morphological diversity, with dozens of extinct and living taxa of cursorial, semiaquatic, scansorial, arboreal and burrowing habits [2,3,5]. Among them is *Hydrochoerus hydrochaeris*, the largest living rodent, reaching an adult weight averaging 40–60 kg [6]. Some extinct species attained even larger sizes, such as *Neoepiblema acreensis* with about 80 kg within a clade encompassing the extant taxa chinchillas and pacaranas. Even larger species include *Phoberomys* spp. and *Josephoartigasia monesi* [2,7–9].

**Figure 1.**
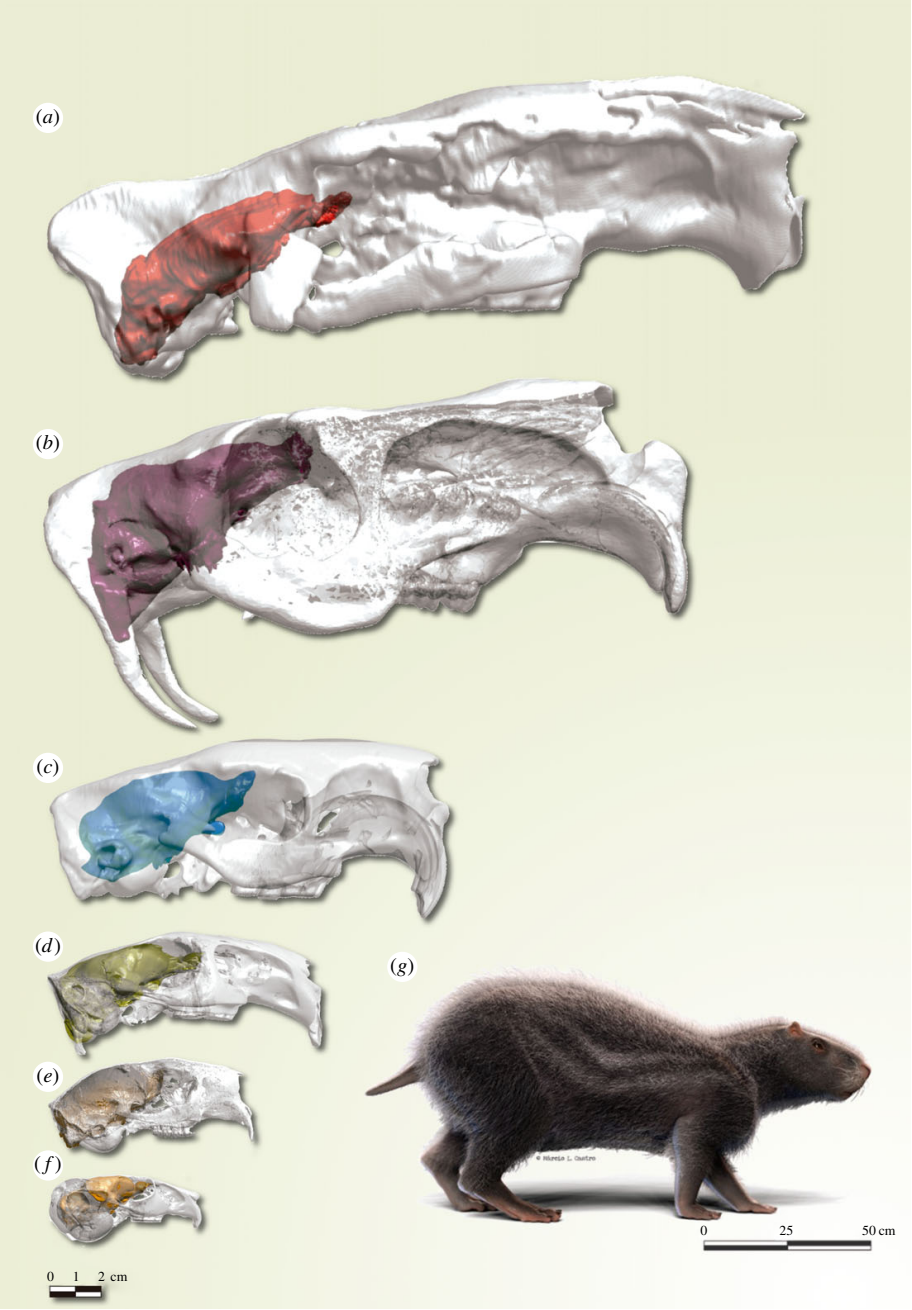
Virtual brain endocast inside of the translucent skull of *Neoepiblema acreensis* (UFAC 4515) from the Upper Miocene of Brazil (*a*) and extant caviomorphs: (*b) Hydrochoerus hydrochaeris* (OUVV 10698); (*c*) *Dinomys branickii* (MCN-D 074); (*d*) *Lagostomus maximus* (CAPPA/UFSM-AC); (*e*) *Coendou spinosus* (MCN 355) (*f*) *Chinchilla lanigera* (OUVC 9529); (*g*) an artistic reconstruction of *N. acreensis* (by Márcio L. Castro).

The impressive ecomorphological diversity and size range of caviomorphs might be expected to have been coupled to neurosensory adaptations reflected in gross anatomical features and brain size. Inferences on the brain size and energetic costs of extinct rodents have been made [10], but in fact, no data on the endocranial anatomy of giant rodents are available. This study uses non-invasive imaging techniques [11] on an exceptionally preserved giant caviomorph skull in the context of a broad examination of endocranial anatomy and size in this group and serves to address the evolution of the brain in this large rodent clade characterized by exceptional size range in the fossil record (figure 1).

## 2. Material and methods

Two specimens from the State of Acre, Brazil (Niteroi site, Upper Miocene Solimões Formation) were analysed: UFAC 4515 (figure 1*a*) is the most complete skull of *Neoepiblema acreensis* known [12,13], while UFAC 3576 is a cranial roof fragment (frontal and parietal). UFAC 3576 preserves the anterior region of the brain endocast, showing impressions of the olfactory bulbs and the frontal and temporal lobes on the internal surface of the cranium (electronic supplementary material, figure S1).

The brain endocast of *N. acreensis* was compared to digitally extracted ones from skulls of the extant and extinct rodents of the four main lineages of Caviomorpha (electronic supplementary material, table S1). For volumetric and further comparisons, data from the literature were also used ([14], electronic supplementary material, table S2). This combination of data resulted in a dataset of 23 hystricognath taxa that could have their encephalization quotients (EQs) calculated.

The skulls analysed in this study were scanned using a medical CT scanner for the large specimens and a Micro CT scanner for the small (less than 10 cm of length) specimens (electronic supplementary material, table S1). The segmentation and generation of the 3D models were performed using Avizo 8.1.0 [15]. The endocranial cavity of *N. acreensis* (UFAC 4515) was manually segmented slice by slice, using a digital tablet, following the osseous boundaries between matrix and bone. The brain endocasts of the extant specimens were generated using both manual and automatic segmentation. Once models were fully reconstructed, volumetric and linear measurements were obtained. The brain endocast of *N. acreensis* is available in MorphoMuseum [16].

EQs were calculated using three different equations for Ec [17–19] following protocols for rodents previously described (e.g. [14]). Additional methodological details are available in the electronic supplementary material.

## 3. Results

### (a) Anatomy

The olfactory bulbs in the UFAC 3576 specimen are well delimited dorsally, slightly elongated and oval-shaped, with a short and well-marked circular fissure (electronic supplementary material, figure S1). They extend anteriorly up to the level of the posterior wall of the M3 and are above the level of the dorsal region at the top of the braincase shown in the occlusal plane in lateral view (figure 1). In *N. acreensis* and *Hydrochoerus hydrochaeris*, they occur at the level of the dorsal region of the cerebral hemispheres, differing from other extant taxa and the extinct *Neoreomys australis* (electronic supplementary material, figures S2–S8) which have the cerebral hemispheres level above that of the bulbs.

The brain endocast of *N. acreensis* shows a separation between the olfactory bulbs and the cerebral hemispheres. The sagittal sinus is continuous throughout the cerebral hemispheres. The rhinal fissure is visible laterally, and the orbitotemporal canal is not visible on the lateral or ventral surface (electronic supplementary material, figure S2).

The cerebrum cast of *N. acreensis* is similar in shape to that of *Dinomys branickii* (electronic supplementary material, figure S7), with the frontal lobes more laterally expanded than in other chinchilloids and erethizontids [20–22] (electronic supplementary material, figure S4B). However, in erethizontids, this character is more apparent, present in fossils since the Early Miocene [22].

As evident in the endocast of UFAC 4515, the cerebellum cast is narrower than the cerebral hemispheres. The telencephalon of *N. acreensis* covers the anterior regions of the cerebellum. The cerebellum is clearly outlined by three parts: a small central vermis and two lateral and more prominent cerebellar hemispheres. The vermis is represented by a small bounded region and separated from the cerebellar hemispheres by paramedian fissures. In Palaeogene forms such as *Ischyromys typus* and *Paramys copei*, and also possibly in the endocast of an Early Miocene ‘cephalomyid’ described by Dozo [22], the cerebral hemispheres do not fully cover the midbrain, exposing this region and distinguishing them from all analysed caviomorphs. Dozo [22] argued that the traits present in ‘cephalomyids’ and other Palaeogene rodents, such as the midbrain dorsally exposed, the absence of well-marked neocortical sulci and the cerebellum with a large vermis, are all plesiomorphic traits in mammals. The brain of *N. acreensis* is more derived than their Early Miocene close relatives ‘cephalomyids’ (electronic supplementary material, figure S9). The paraflocculus is not evident in the brain endocast of *N. acreensis* (see electronic supplementary material, figure S2). Among the caviomorphs analysed, in those specimens without evident paraflocullar lobes, the telencephalon covers the cerebellum to a higher degree than in those that have well-developed paraflocculus. In both brain endocasts, the intracranial dural sinus system (sagittal dorsal sinus and transversal sinus) is visible. It forms a superior sagittal sinus that covers the entire cerebral hemispheres surface and is continuous with the transverse sinus at its posterior end. The sagittal dorsal sinus is well marked and protruding.

### (b) Encephalization quotient and scaling

The brain endocast volume of *Neoepiblema acreensis* is 49 682.06 mm³. This value is probably just below the real value because of the bias taphonomic loss of information. The EQs of UFAC 4515 were calculated using three different equations. The EQs were calculated following Jerison [17] [EQ = EV/(0.12 × BM^0.67^)], Eisenberg [18] [EQ = EV/ (0.0553 × BM^0.74^)] and Pilleri *et al*. [19] [EQ = EV/(0.00997 × BM^0.6419^)]. Using the average of the weight estimates as a proxy for body mass, the EQs of *N. acreensis* are 0.20 [17,18] and 0.33 [19]. These values are low compared with those of other rodents (electronic supplementary material, table S3).

It is not possible to know exactly how much volume the endocranial cavity lost during the diagenetic processes. To account for the potential effect of taphonomy, in particular, a potential volume loss, we arbitrarily added 10–40% in volume brain to test the effect of this increasing on EQ values. Even with this artificial increase in volume brain, the EQs are still low (electronic supplementary material, table S4). For the sake of comparison, the largest living rodent *Hydrochoerus hydrochaeris* has an average EQ of about 1.01, based on the equation of Pilleri *et al*. [19]. To obtain a similar relationship between brain and body mass (EQ), the endocranial volume of *N. acreensis* should be increased by 300%.

On applying the scaling equation of Herculano-Houzel *et al*. [10], the estimated brain mass of *N. acreensis* was 114 g. On the other hand, converting the volumetric information of the endocranial cavity of UFAC 4515 to brain mass, the result is 47.31 g.

## 4. Discussion

**Figure 2.**
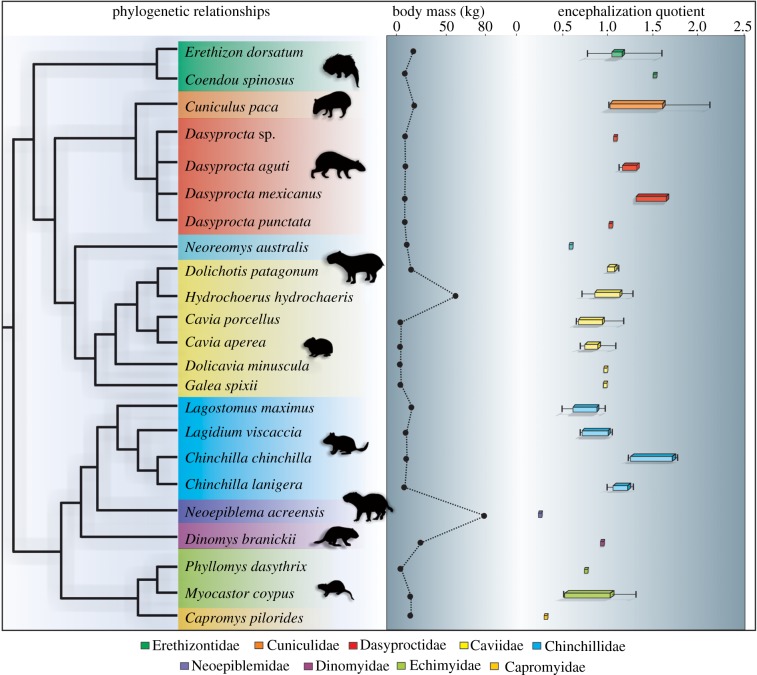
Encephalization quotient and body mass in Caviomorpha [17]. The composed phylogeny is based on Upham & Patterson [3]. The phylogenetic position of *N. acreensis* is based on Kerber *et al*. [13] and Rasia & Candela [7], and *N. australis* and *Dolicavia minuscula* on Pérez *et al*. [23]. The EQs are based on this work (electronic supplementary material, table S3) and Bertrand & Silcox [14] (index electronic supplementary material, table S3). The box plot of the EQ of *N. acreensis* includes the whole spectrum of estimates of endocranial volume, including corrections for potential taphonomic bias ranging from 10 to 40% of the primary volume (electronic supplementary material, table S4).

*Neoepiblema acreensis* is one of the largest rodents that inhabited South America. This rodent shows a low EQ compared with other hystricognaths (electronic supplementary material, table S2; figure 2). The allometric analysis of the brain and body mass of the extant and extinct representatives of the four caviomorph clades examined demonstrates that *N. acreensis* has a smaller brain mass than expected for its body mass compared with extant chinchilloids (electronic supplementary material, figure S10). Even assuming that the brain endocast lost part of its original volume (electronic supplementary material, table S4), the EQs are still low compared to those of other rodents (figure 2).

The encephalization of extant caviomorph representatives of all less inclusive clades (families) has been studied (see [14]; electronic supplementary material, table S2). Most of them show an EQ average above 1.05 [14]. One of the extant caviomorphs with the lowest EQ is *Capromys pilorides* (0.39; electronic supplementary material, table S2), which inhabits Cuba and nearby islands [24] where large mammalian predators are absent. Our EQ knowledge of extinct caviomorphs is quite limited. Besides the neoepiblemid here studied, EQs are available for two other Neogene small-/medium-sized species. The Early Miocene *N. australis* (electronic supplementary material, figure S8) has an estimated EQ of 0.52, while the Late Pliocene extinct caviid *Dolicavia minuscula* shows a high EQ (1.02) [21], similar to its extant Cavioidea close relatives (1.02).

Herculano-Houzel *et al*. [10] presented scaling rules for the allometric relationships between brain and body mass in extant rodents. They predicted the brain mass of giant extinct rodents and concluded they could have had large brains, but considerably fewer neurons compared to primates of similar mass. A large brain can bring benefits while also imposing higher energetic costs, leading Herculano-Houzel *et al*. [10] to hypothesize that the large brains of giant rodents could have been physiologically costly and may have contributed to their extinction. Based on a body size estimate of 79.75 kg (average of the estimates in electronic supplementary material, table S5), we applied brain mass scaling rules [10] to *N. acreensis*, which resulted in an estimated brain mass of 114 g––almost three times higher than the values calculated from the endocast of UFAC 4515 (electronic supplementary material, table S3). The brain mass estimate based on extant Glires [10] is higher than the values obtained from the endocast here studied, even considering the 30% error margin in the prediction model for extinct forms. The largest known extinct caviomorph, *Josephoartigasia monesi,* possibly had a relatively small brain mass for its size, as indicated by the sagittal cranial section in Rinderknecht & Blanco [9] where it is possible to see a small endocranial cavity when compared with the total size of the skull.

The presence of relatively small brains in mammals can be associated with a temporal effect in brain size (i.e. ‘primitiveness’) [17], since several groups of mammals show an increase in encephalization from the basal to derived forms [25–28], which apparently does not occur in rodents due to their high taxonomic and ecological diversity [14,28]. Alternatively, small brains can be related to evolutionary processes such as fossoriality, due to the lack of visual specialization (and other traits) [29], changes in social behaviour [30], domestication [31] and insularism [32,33] (exemplified in caviomorphs by *C. pilorides*), processes that are associated with the absence/reduction of predators and low competition (e.g. ‘Red Queen effect’) [34,35].

Although neoepiblemids did not evolve on small islands, between the Oligocene (when the Drake Passage was formed) and the Late Miocene/Early Pliocene (when the Isthmus of Panama was formed), the South American continent was isolated. This isolation may have led to evolutionary results similar to those associated with insular processes, for example, in generating several lineages of rodents of large body mass, a pattern not present in other continents [36]. Additionally, in the absence of large placental carnivores (active predators), the likeliest predators to large rodents of the tropical region of South America were large crocodilians [37], which were probably sit-and-wait strategists. This hypothetical trophic scenario implies different predation pressures [35,38] during the Late Miocene as compared to those in South American environments after the arrival of the placental carnivores and could have influenced palaeoneurological adaptation.

In conclusion, the analysed large rodent in this study had a low EQ compared to other forms, including other Neogene medium-sized caviomorphs (*N. australis* and *D. minuscula*). Without the benefits of a high density of neurons, a large brain implies unnecessary energetic costs when associated with large bodies that are also metabolically costly [10]. Thus, without ecological pressure (see above), there would be no need to increase brain size in these large Neogene rodents. In the absence of data (i.e. EQs) of other extinct forms basal to neoepiblemids, it is not possible to know at this time if the low EQ is a plesiomorphic pattern maintained during the Late Miocene or if they reduced it secondarily. The ancestral character reconstruction shows that the ancestor pattern of EQ for the analysed caviomorphs is between 0.94 and 1.06 (electronic supplementary material, figure S11). However, this estimate is mostly based on extant forms; the inclusion of more data of extinct species is needed to analyse this aspect.

## Supplementary Material

Supplementary information

## Data accessibility

The surface renderings of the brain endocasts of *Neoepiblema acreensis* are available on MorphoMuseuM http://morphomuseum.com/Specimenfiles/sendFile/502/395e46.
