# Supplementary material for: Small within the largest: brain size and anatomy of the extinct Neoepiblema acreensis, a giant rodent from the Neotropics
Source: Biol Lett. 2020 Feb 12;16(2):20190914. doi: 10.1098/rsbl.2019.0914 (PMC7058952; doi:10.1098/rsbl.2019.0914)
Supplement: Supplementary information [file rsbl20190914supp1.docx]

SUPPLEMENTARY INFORMATION

**Small within the largest: Brain size and anatomy of the extinct *Neoepiblema acreensis*, a giant rodent from the Neotropics**

José D. Ferreira^1^, Francisco R. Negri^2^, Marcelo R. Sánchez-Villagra^3^, Leonardo Kerber^1,4^

1. Programa de Pós-Graduação em Biodiversidade Animal, Universidade Federal de Santa Maria, Santa Maria, Brazil
2. Laboratório de Paleontologia, Campus Floresta, Universidade Federal do Acre, Cruzeiro do Sul, Acre, Brazil
3. Palaeontological Institute and Museum, University of Zurich, Zurich, Switzerland
4. Centro de Apoio à Pesquisa Paleontológica da Quarta Colônia (CAPPA), Universidade Federal de Santa Maria, São João do Polêsine, Brazil.

Authors for correspondence:

leonardokerber@gmail.com

darival.ferreira@gmail.com

*
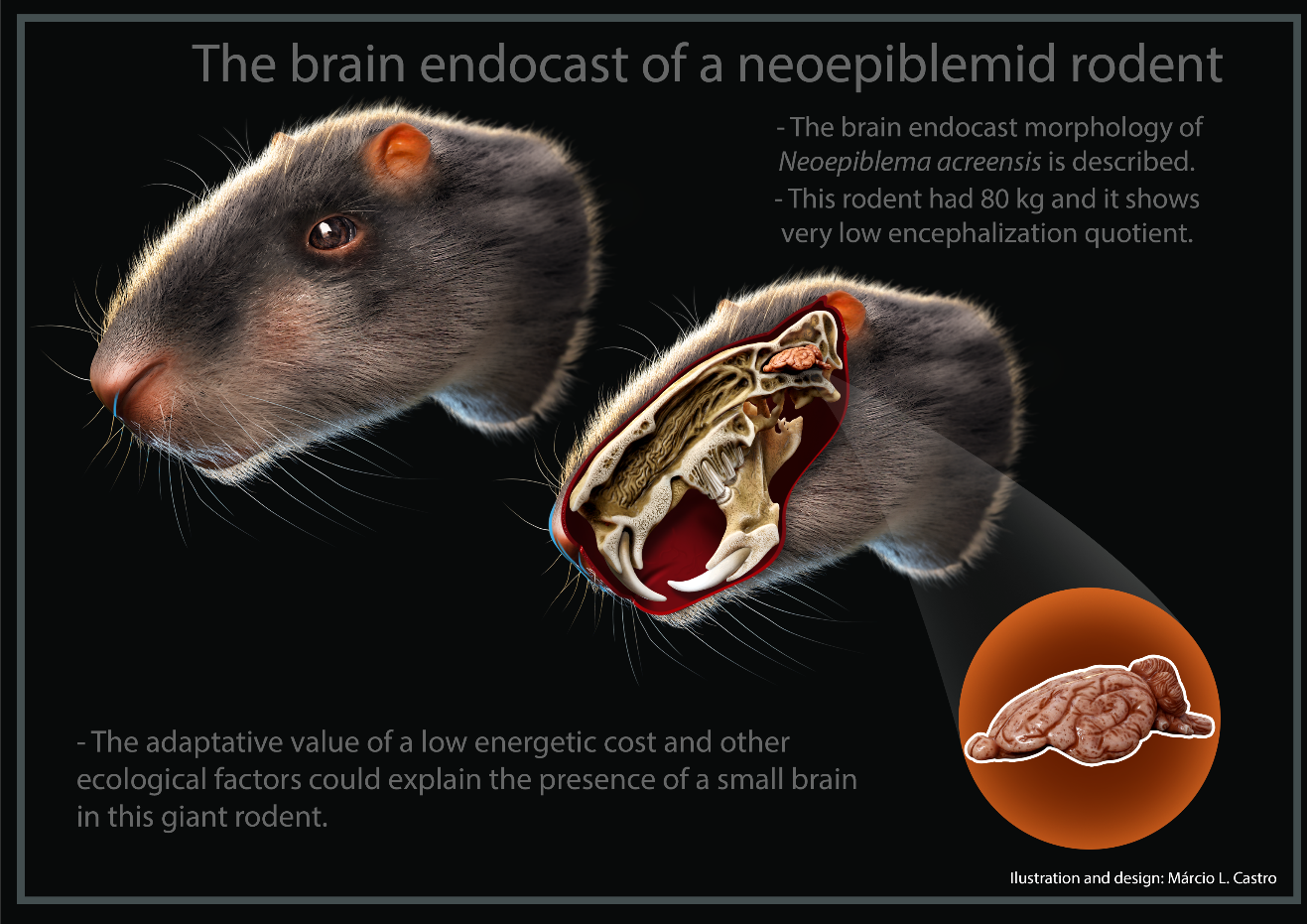
*

**Graphical Abstract**: Rodentia is the most diverse group of extant mammals, and some extinct South American members of this clade reached giant body size during the Late Miocene, some 10 million years ago. The study of the anatomy and size of exceptionally preserved giant caviomorph skull in the context of a broad examination of other rodents demonstrates that in opposition to their large body mass, these extinct rodents had a small brain when compared to the extant forms. The adaptive value of a low energetic cost and other ecological factors are possibly associated with the relative small brain size of giant rodents.

**Material and Methods**

UFAC 4515 shows some missing regions, such as the anterior region of the right nasal, and some fractures on the dorsal and lateral regions of the skull. The skull also underwent diagenetic modifications, which resulted in a slight distortion of the original shape. On the other hand, the braincase is preserved, which permitted access to the general morphology of the brain endocast.

For comparative purposes, we used CT-Scans of other caviomorphs (Table S1). The specimen of *Neoreomys australis* (PIMUZ A/V 5265) was described in Zurita et al. [1]. The scans of *Cavia porcellus* and *Dasyprocta* sp. (originally identified as *Dolichotis patagonum -* www.digimorph.org/specimens/Dolichotis_patagonum *-* but here treated as *Dasyprocta* sp.) were downloaded from digimorph.org. The scans of *Hydrochoerus hydrochaeris* and *Chinchilla lanigera* were kindly provided to us by Lawrence Witmer (https://people.ohio.edu/witmerl/lab.htm). Other specimens were scanned for this study.

To access the endocranial information of UFAC 3576, besides the direct observations on the bone surface, a brain endocast of the specimen UFAC 3576 was generated using silicone rubber.

***Linear measurements, encephalization, body mass estimates***

The linear measurements of the brain endocasts were taken following [2] (Table S6). The encephalization quotient (EQ) is a measure of relative brain size [3]. The EQ is the ratio of the actual brain size of a species and the expected brain size for a "typical" hypothetical mammal of the same body mass. The EQs were calculated using three different equations for Ec [3–5] following previous protocols for rodents (e.g. [6]).

The body mass of *Neoepiblema acreensis* and *Neoreomys australis* were calculated following the equations developed by Millien [7] based on eight variables to *N. acreensis* (Table S5) and three variables to *N. australis* (Table S7). To calculate the EQs of the comparative specimens, we used the body mass data available in the literature (average) (Table S5).

The linear regression (M_BR_ = 0.037 x M_BD_^0.712^) to predict the brain mass based on the body mass developed by Herculano-Houzel et al. [8] was employed to test if predictions based on the data of living rodents reflect the brain mass estimates obtained from endocasts.

The brain endocast of *N. acreensis* was reconstructed based on CT-Scan, as well as a silicone rubber model, using the specimens UFAC 4515 and UFAC 3576, respectively. It was possible to observe the cast of several structures and compare them with other caviomorphs, such as olfactory bulbs, cerebrum, cerebellum, veins, and cranial nerves.

***Ancestral character state reconstruction***

To analyze the phylogenetic distribution of the Pilleri et al. [5] EQs, as well as, to reconstruct their ancestral condition, these values were mapped as a continuous character in a composed phylogeny of Caviomorpha using the software Mesquite 3.03 [9]. When the EQs showed individual variation (see Table S2), we used their mean value. The topology is based on Upham and Patterson [10], who analyzed DNA sequences of nuclear genes. Extinct taxa (*Neoreomys australis*, *Dolicavia minuscula*, and *Neoepiblema acreensis*) were included in a sister-group relation with the closer living taxon, based on cladistic hypotheses [11,12]. The function Trace Character History was employed to reconstruct the ancestral states using the Parsimony Ancestral State method [9]. *Hystrix cristata*, *Paramys* spp. and *Ischyromys typus* were used as outgroups.

**Institutional abbreviations**

**AMNH,** American Musem of Natural History, USA; **CAPPA/UFSM-AC**, Comparative anatomy collection of the Centro de Apoio à Pesquisa Paleontológica da Quarta Colônia/Universidade Federal de Santa Maria, Brazil; **MCN-M** and **MCN-D**; mammal collection of the Fundação Zoobotânica do Rio Grande do Sul, Brazil; **OUVC,** Ohio University Vertebrate Collections, USA; **TMM**, Texas Memorial Musem, USA; **PIMUZ** **A/V**, Paleontological Institute and Museum, University of Zurich; **UFAC,** paleontological collection of the Universidade Federal do Acre (Campus Rio Branco), Brazil.

**Extended anatomical description and comparison**

***Olfactory bulbs***

The olfactory bulbs of the specimen UFAC 4515 are deformed, and it is not possible to access their morphology in detail. On the other hand, the specimen UFAC 3576, which corresponds to the cranial roof (frontal and parietal), has the dorsal impression of the bulbs well preserved. The olfactory bulb cast has a 12.2 mm width x 15.8 mm length.

The portion of the olfactory bulbs of the brain endocasts of some caviomorphs shows some differences in comparison to the real brain. For example, the real olfactory bulbs of *H. hydrochaeris* are ovoid (see [13]), while the cast has an irregular shape (Figure S3a).

***Nerves and Vessels***

The portion of the peduncles of the left optic nerve is located on the ventral region of the anteriormost portion of the brain endocast. Posterior to the optic nerve, is the impression of the sphenoidal fissure (=anterior lacerate foramen), which transmits the internal maxillary artery [14], as well as cranial nerves III, IV, V (variable V2), and VI [15].

Between both middle lacerate foramen + the foramen ovale is the impression of the pituitary fossa, which is damaged by slight ventral compression of the skull.

On the posterior region of the endocast is the cast of the jugular fossa (see [16]) or posterior lacerate foramen, which is an oblique impression. This impression represents the carotid foramen anteriorly and posteriorly the jugular foramen (Figure S1). In rodents, the jugular fossa conveys the internal jugular vein, carotid, and stapedial arteries, as well as cranial nerves IX, X, and XI [17]. Laterally to these structures is the internal acoustic meatus (passage for the nerves VII and VIII), and posteriorly is the impression of the hypoglossal nerve (XII).

***Cerebrum***

The deep lateral sulcus longitudinally extends from the posterior portion to the anterior portion of the neocortex, terminating anteriorly in a transverse convolution. Anteriorly to this convolution, there is a sulcus, in the same topographic location of the ansate sulcus found in other mammals [18]. The other sulci and convolutions are also well marked on the brain endocast. The suprasylvian sulcus is more visible dorsally, but it is not as deep as the lateral sulcus, as well as the ectosylvian sulcus.

The cerebrum cast of *N. acreensis* is similar in shape to that of *D. branickii* (Figure S7)*,* showing the frontal lobes more developed than in other Chinchilloidea, and in erethizontids (see [19,20]). However, in erethizontids, this trait is more evident, being present in fossils since the Late Miocene [19]. On the other hand, the frontal lobes of *N. acreensis* of both specimens analyzed seem to have a dorsoventral compression more evident than in the other caviomorphs examined (Figure S3-S4).

The cerebrum of *N. acreensis* shows a girencephalic pattern similar to those of *L. maximus* (Figure S6), *D. branickii* (Figure S7), and *H. hydrochaeris* (Figure S3a)*.* The neurocortical patterns of the cortex of caviomorph rodents are quite diverse, varying from smooth without furrow or convolutions (lissencephalic), to fold in numerous convolutions (girencephalic) [20,21]. Pirelli et al. [5] proposed a correlation between the development of neocortical sulcus and body size. Dozo [22] demonstrated that in the larger forms of Chinchillidae, Caviidae, and Dasyproctidae, there is a greater complexity in the neocortical sulci than in the smaller forms. The caviids, for example, present well-defined body size patterns, which correlate with the complexity of the neocortical sulci patterns [21].

The lateral sulcus of the brain endocasts chinchilloids is deeper and more marked than in the other caviomorphs. In *N. acreensis,* the lateral sulcus is not continuous anteroposteriorly through all the extension of the neocortex, like *D. branickii*. The brain endocasts of *N. acreensis* and *D. branickii* have the continuity of the lateral sulcus interrupted by a sulcus similar to the ansate sulcus. Madozzo-Jaén [21] observed that in most Caviidae, the lateral sulcus extends throughout the neocortex extension, but not in *Cavia porcellus*, *Dolicavia minuscula*, and *Dasyprocta primnolopha*. In the specimen of *Dasyprocta* sp. analyzed here, the lateral sulcus is present and continuous throughout the extension, indicating a variable character among caviioids.

The intracranial dural sinus system in *N. acreensis* forms a superior sagittal sinus that covers all the surface of the cerebral hemispheres. At its posterior end, it is continuous to the transversal sinus. These same prominences are observed in *L. maximus* (Figure S5) and *D. branickii* (Figure S7), although they are not prolonged on the dorsal region of the cerebrum as in *N. acreensis*.

***Cerebellum***

The vermis is smallerthan the cerebellar lobes in *N. acreensis* (Figure S2), different from some rodents such as caviids and octodontoids analyzed here, in which the vermis is more developed than the lobes.

**The brain endocast of *Neoepiblema acreensis* in the context of Chinchilloidea**

Concerning the context of the brain endocast of Chinchilloidea, although the limited knowledge on the extinct forms (Figure S9), it is possible to observe that the small forms, such as ‘cephalomyids’ (which are close to neoepiblemids than to other caviomorphs [22]) and *C. lanigera* show share some plesiomorphic traits, such as a lissencephaly, division of olfactory bulbs, cerebrum, and cerebellum, including a possible dorsal exposition of the midbrain in the case of ‘cephalomyids’. *L. maximus*, which is larger than the both mentioned taxa, shows more circumvolutions on the neocortex, and *D. branickii,* which are the larger analyzed chinchilloids, shows a girencephalic brain, with an expansion of the frontal lobe, and paraflocculus not evident.

# Figures


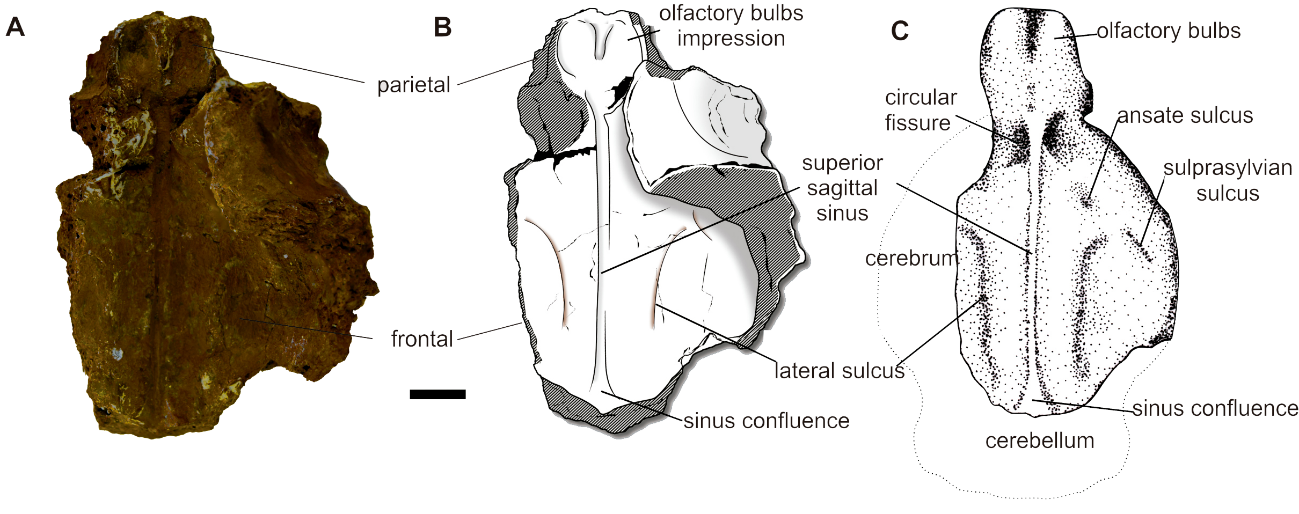
**Figure S1**. **A**-**B**, Skull fragment (frontal and parietal) (UFAC 3576) of *Neoepiblema acreensis* from the Upper Miocene of Brazil, and schematic drawing in ventral view (**A**, **B**, respectively). **C**, brain endocast in dorsal view. Scale-bar: 10 mm.


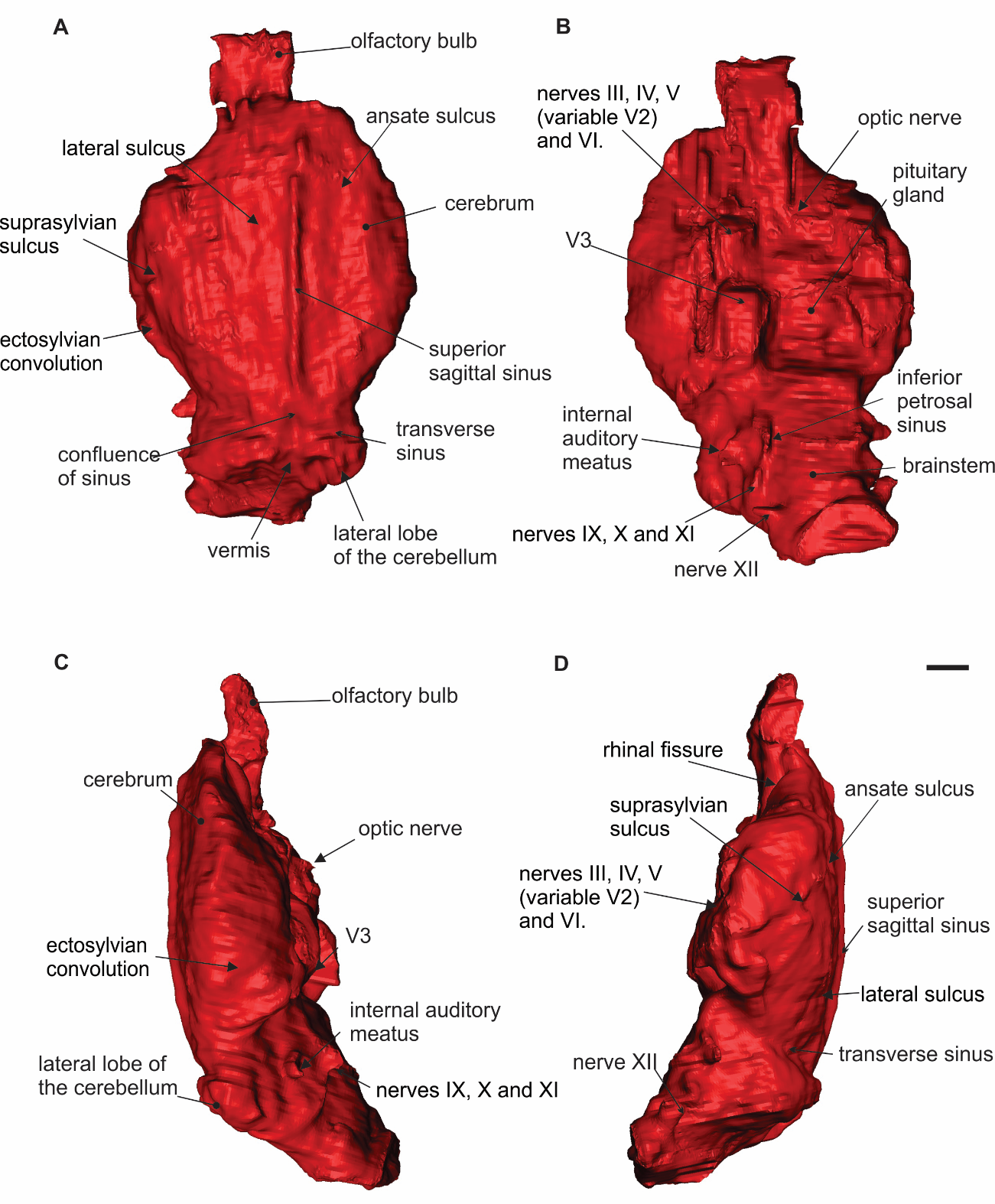


**Figure S2**. Brain endocast of *Neoepiblema acreensis* (UFAC 4515) from the Upper Miocene of Brazil, in dorsal (**A**), ventral (**B**), right lateral (**C**), and left lateral (**D**) views. Scale = 10 mm.


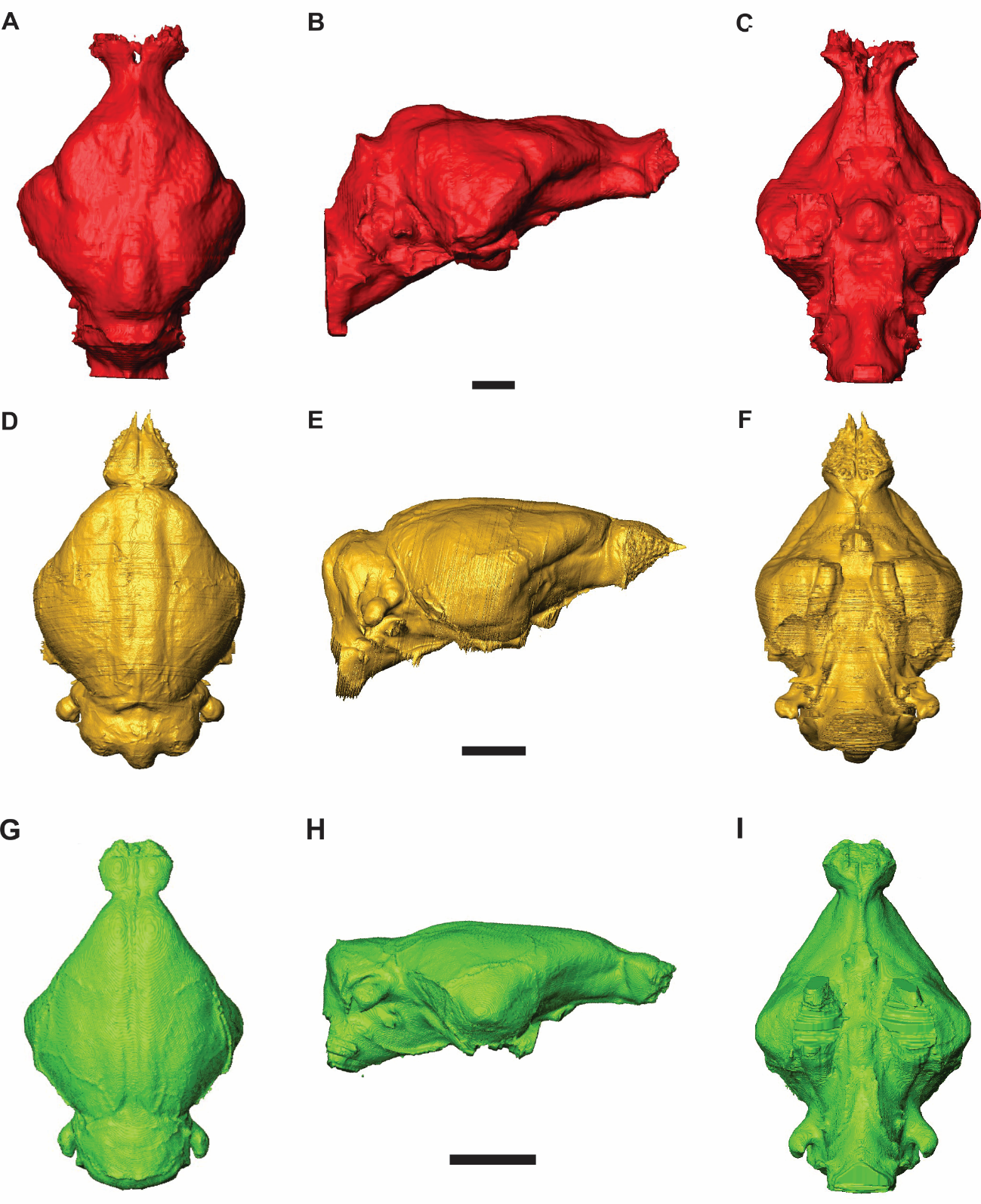


**Figure S3**. Diversity of brain endocast morphology of some extant caviomorphs, in dorsal (**A**, **D**, **G**), lateral (**B**, **E**, **H**), and ventral (**C**, **F**, **I**) views. **A**-**C**, *Hydrochoeerus hydrochaeris* (OUV 10698). **D**-**F**, *Dasyprocta* sp. (AMNH 80078). **G**-**I**, *Cavia porcellus* (TMM M-7283). Scale = 10 mm.


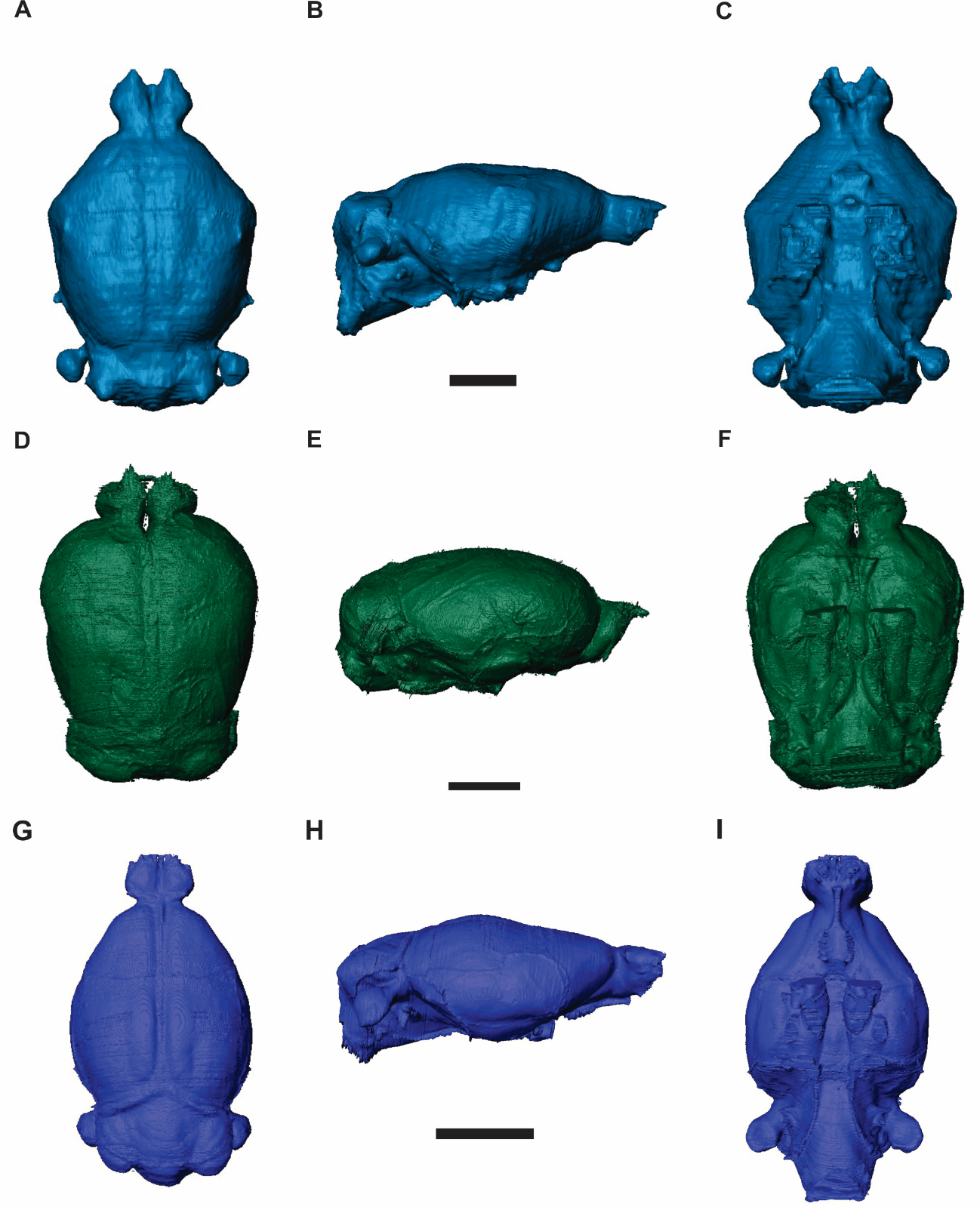


**Figure S4**. Diversity of brain endocast morphology of some extant caviomorphs, in dorsal (**A**, **D**, **G**), lateral (**B**, **E**, **H**), and ventral (**C**, **F**, **I**) views. **A**-**C**, *Myocastor coypus* (CAPPA/UFSM - AC). **D**-**F**, *Coendou spinosus* (MCN-M 335). **G**-**H**, *Phyllomys dasythrix* (MCN-M 391). Scale = 10 mm.


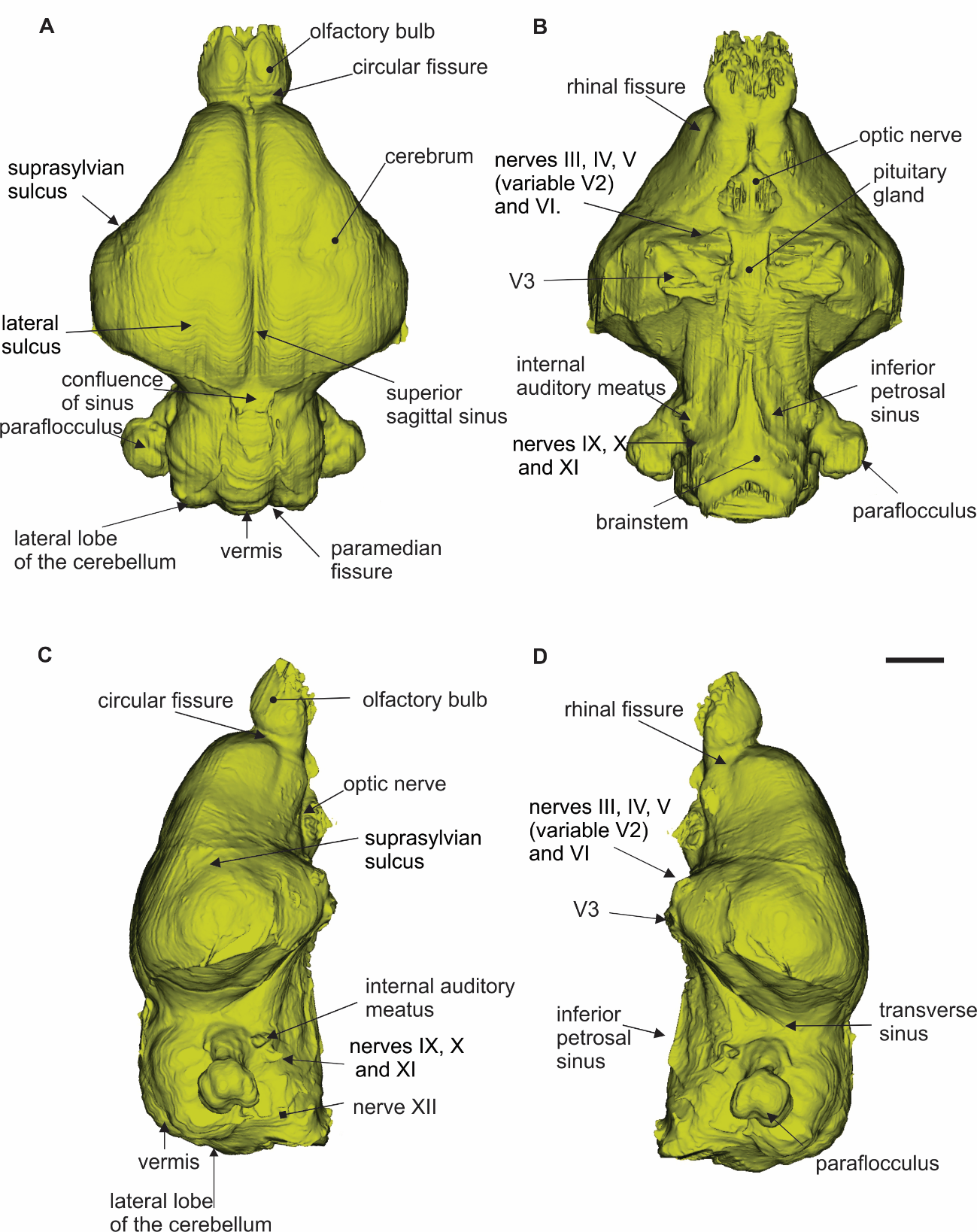


**Figure S5**. Brain endocast of *Chinchilla lanigera* (OUV 9529), in dorsal (**A**), ventral (**B**), right lateral (**C**), and left lateral (**D**) views. Scale = 10 mm.


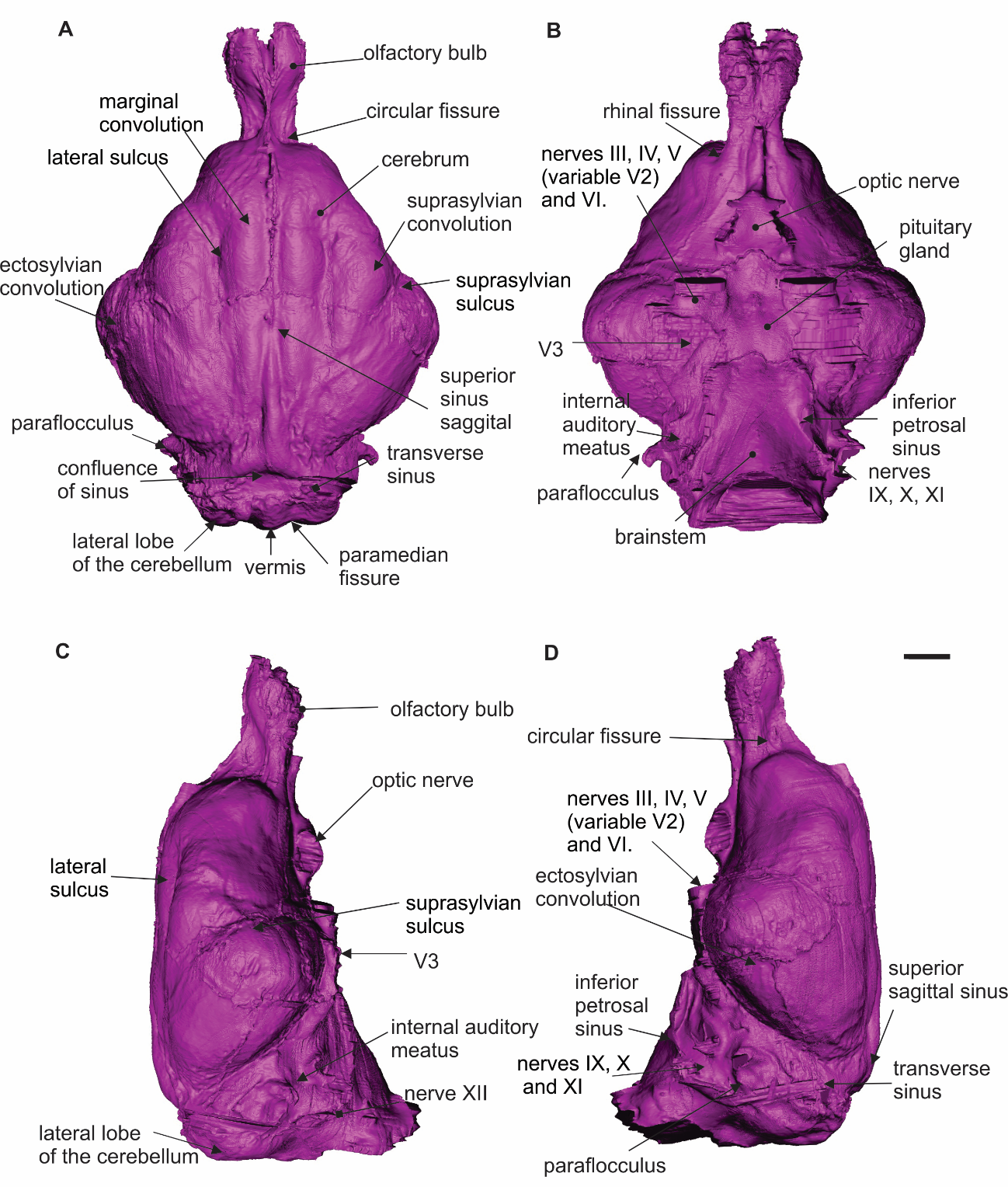


**Figure S6**. Brain endocast of *Lagostomus maximus* (CAPPA/UFSM - AC), in dorsal (**A**), ventral (**B**), right lateral (**C**), and left lateral (**D**) views. Scale = 10 mm.


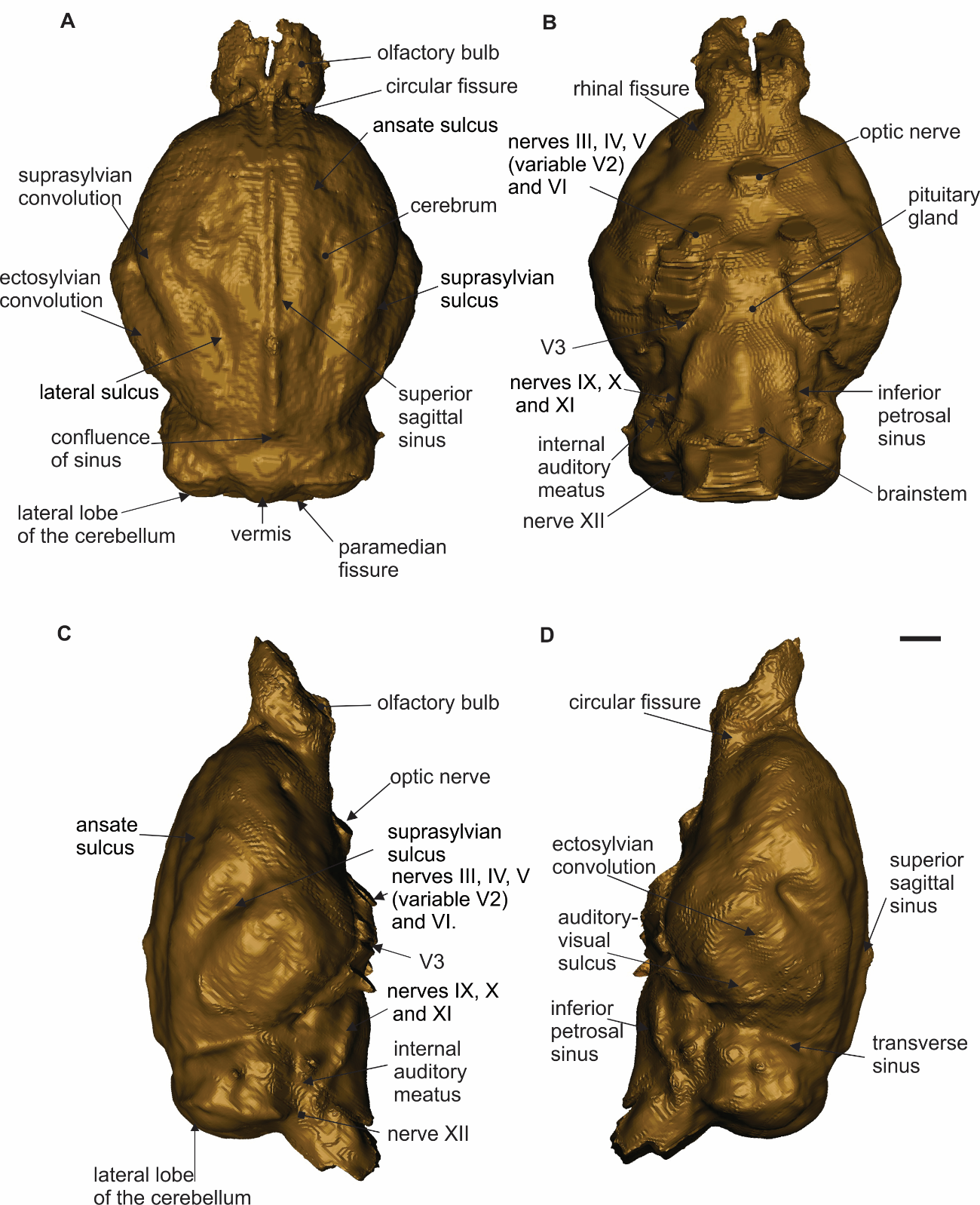


**Figure S7**. Brain endocast of *Dinomys branickii* (MCN-D 072), in dorsal (**A**), ventral (**B**), right lateral (**C**), and left lateral (**D**) views. Scale = 10 mm.


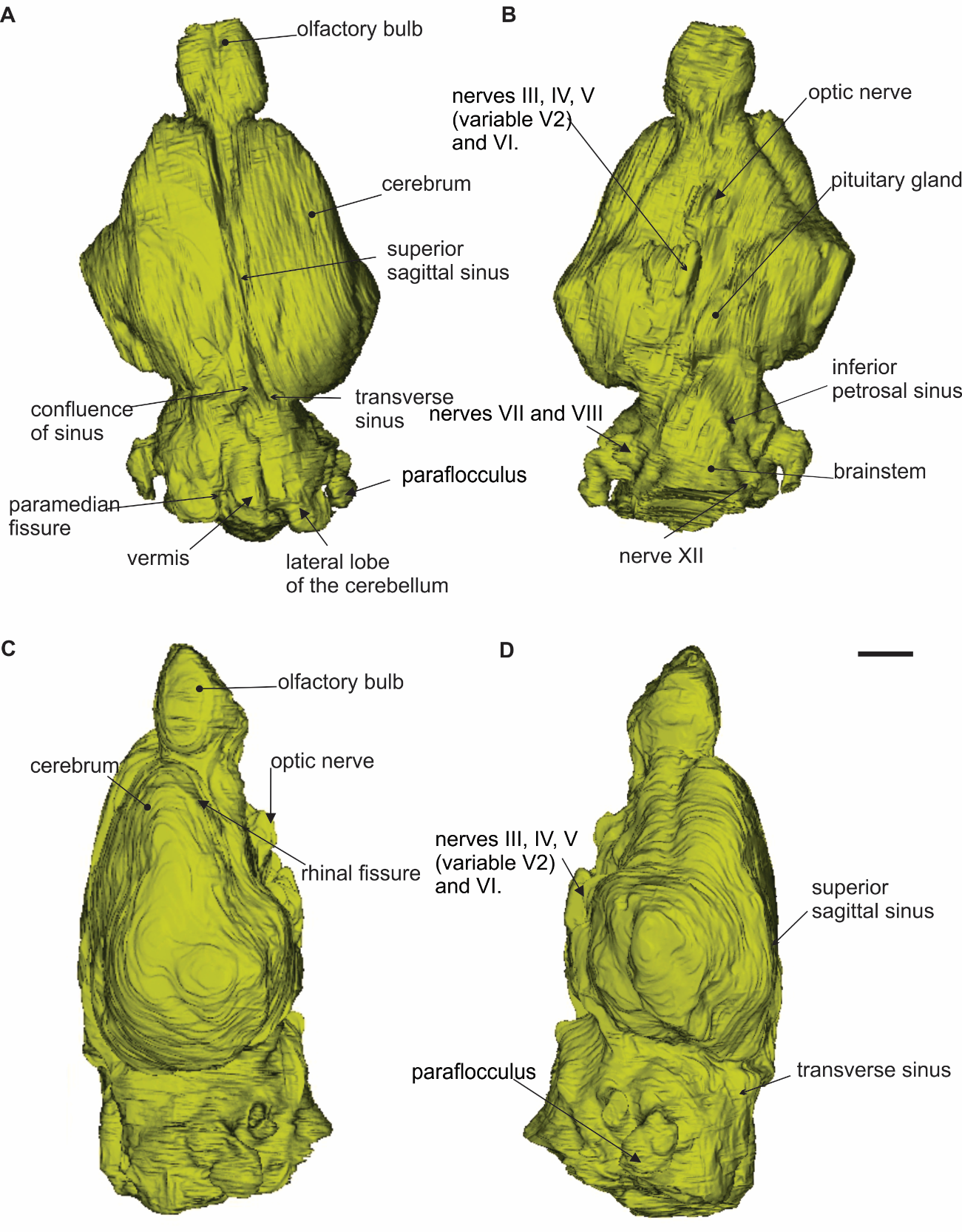


**Figure S8**. Brain endocast of *Neoreomys australis* (PIMUZ A/V 5265) from the Early Miocene of Santa Cruz Formation, Argentina, in dorsal (**A**), ventral (**B**), right lateral (**C**), and left lateral (**D**) views. Scale = 10 mm.


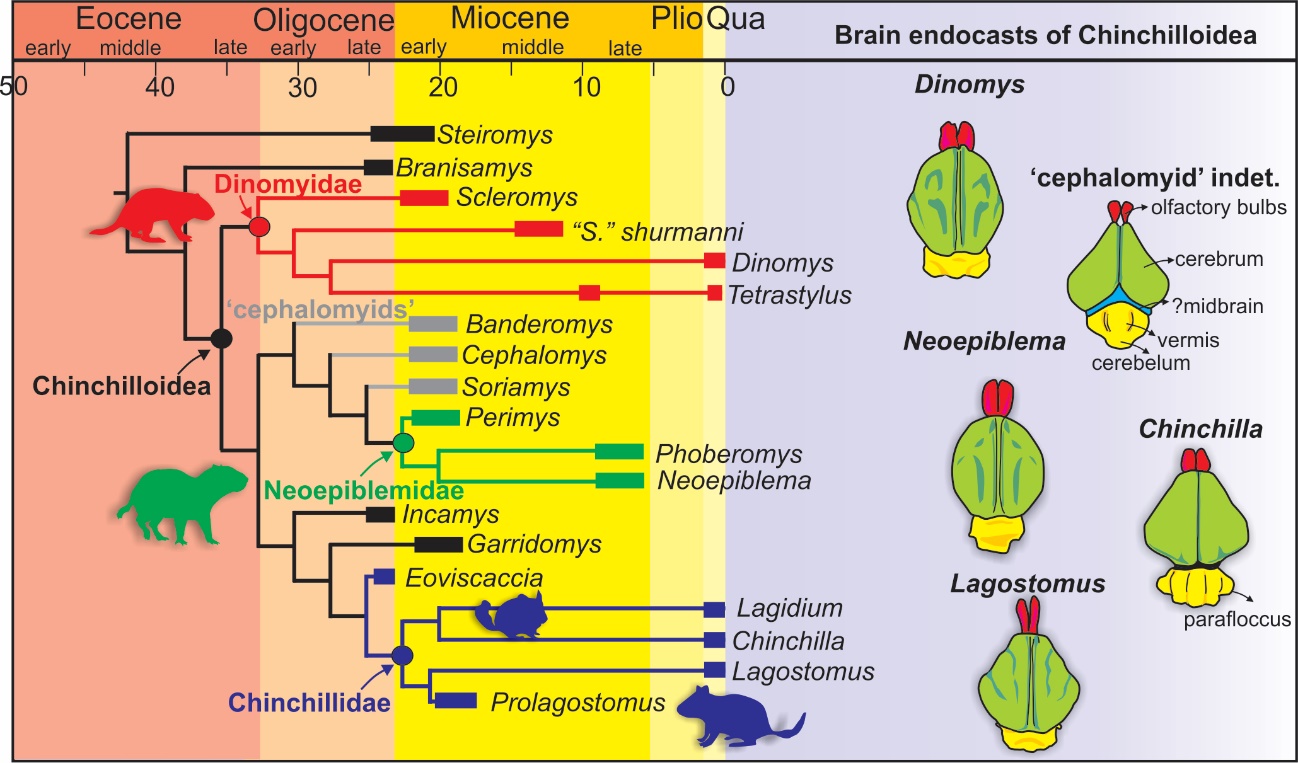


**Figure S9**. Phylogenetic relationships and stratigraphic distribution of Chinchilloidea [23] and brain endocast morphology (based on this work and Dozo [22] for the ‘cephalomyid indet.’).

**
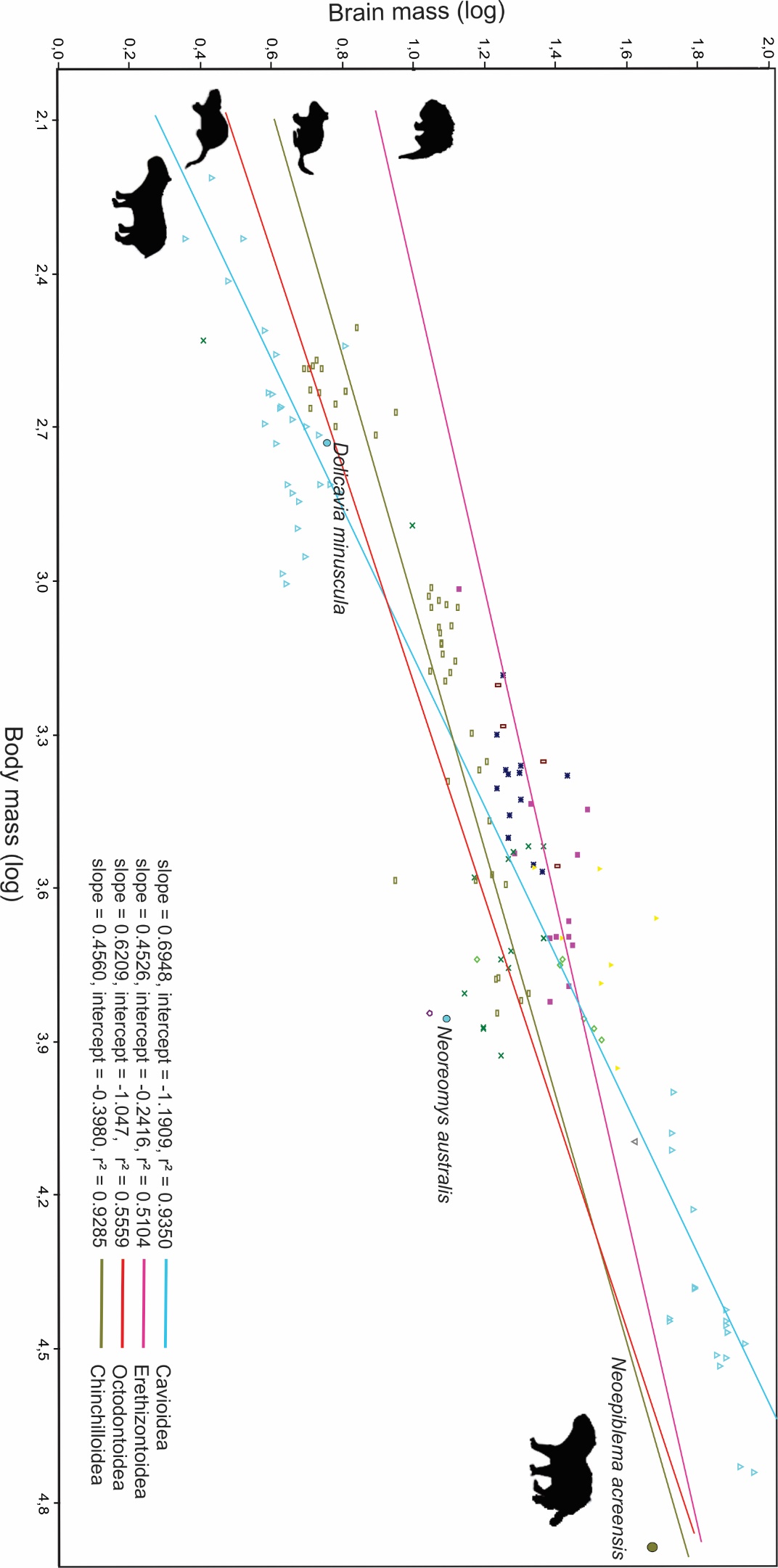
**

**Figure S10.** Bivariate plot of brain and body mass (log) and least squares regression lines of extant Caviodea, Erethizontoidea, Octondontoidea, and Chinchiloidea, plus the extinct *Neoepiblema acreensis*, *Dolicavia minuscula,* and *Neoreomys australis*.


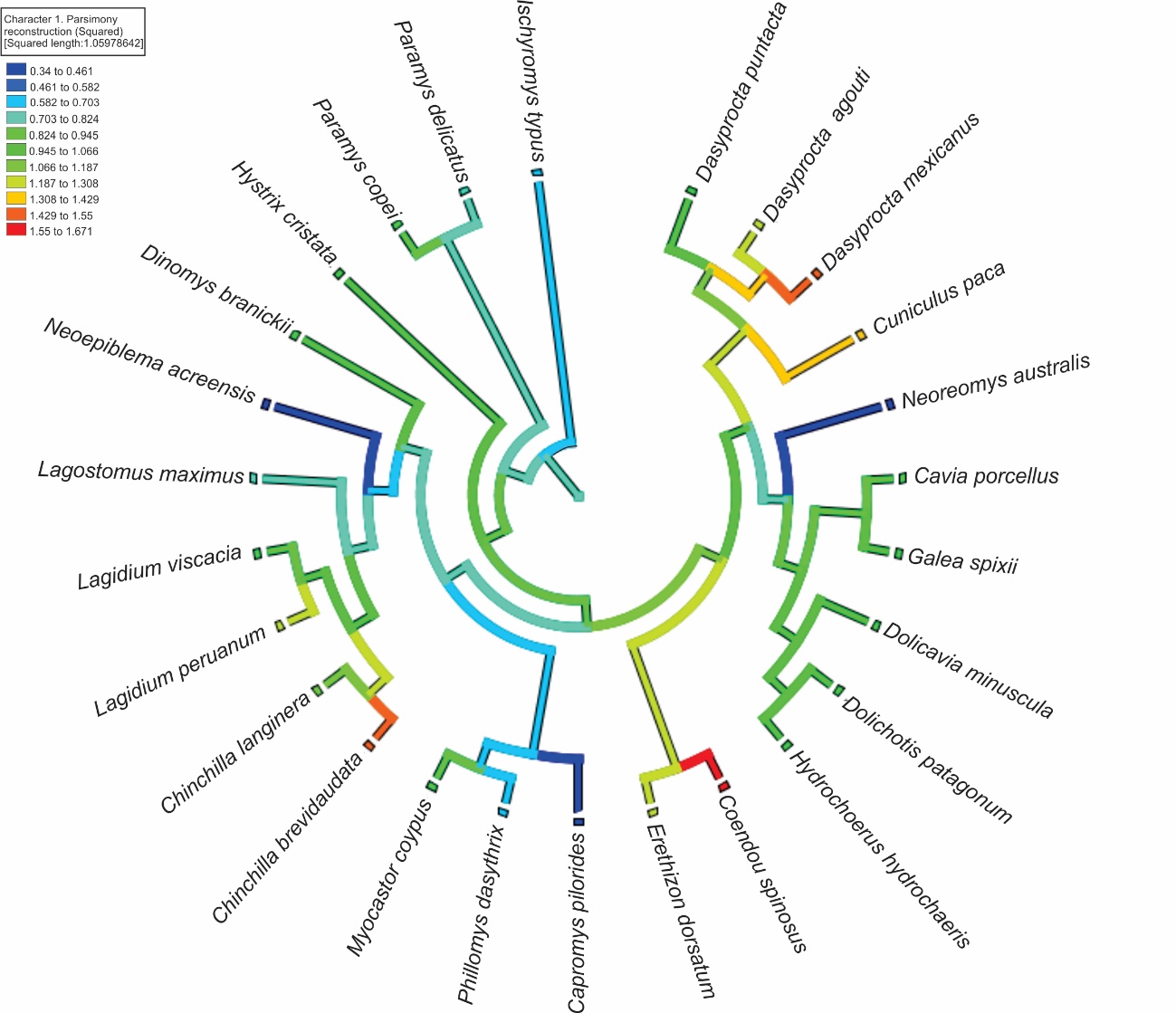


**Figure S11.** Ancestral states reconstruction and evolution of the Pilleri et al. [5] Encephalization Quotient. The composed topology of Caviomorpha is based on Upham and Patterson [10], Pérez et al. [11], and Kerber et al. [24]).

# Tables

**Table S1**. Computed tomography (CT) scan parameters.

| **Species** | **Specimen number** | **Tomograph** | **Voxel Size (mm)** | **Voltage (Kv)** | **Current** |
| --- | --- | --- | --- | --- | --- |
| *Neoepiblema acreensis* | UFAC 4515 | GE Medical | 0.488 | 100 | 223.4 mAs |
| *Neoreomys australis* | PIMUZ A/V 5265 | uCT | 0.058 | 200 |  |
| *Chinchilla lanigera* | OUV 9529 | uCT | 0.046 | - | - |
| *Lagostomus maximus* | CAPPA/UFSM - AC | uCT | 0.035 | 75 | 106mAs |
| *Dinomys branickii* | MCN-D 072 | GE Medical | 0.260 | 120 | 399.84 mAs |
| *Hydrochaerus hydrochaeris* | OUV 10698 | GE Medical | 0.391 | 120 | 132 mAs |
| *Cavia porcellus* | TMM M-7283 | uCT | 0.041 | 180 | 0.25mAs |
| *Dasyprocta* sp. | AMNH 80078 | uCT | 0.058 | 200 | 0.10 mA |
| *Coendou spinosus* | MCN-M 335 | uCT | 0.026 | 110 | 72 uA |
| *Myocastor coypus* | CAPPA/UFSM - AC | GE Medical | 0.195 | 140 | 150 mAs |
| *Phyllomys dasythrix* | MCN-M 391 | uCT | 0.024 | 65 | 1202uA |

**Table S2**. Dataset on brain and body masses, and Encephalization Quotients of comparative rodents, with emphasis to Hystricognathi. The dataset is modified from Bertrand and Silcox [2] (Table S3), with the addition of the rodents analyzed here.

| **Species** | **Brain mass (g)** | | **Body mass (g)** | | **EQ Jerison** | **EQ Eisemberg** | | **EQ Pilleri et al.** | |
| --- | --- | --- | --- | --- | --- | --- | --- | --- | --- |
| *Ischyromys typus* [2] | 5.31 | | 1401.8 | | 0.35 | 0.45 | | 0.51 | |
| *Ischyromys typus* [2] | 5.65 | | 1086.42 | | 0.44 | 0.58 | | 0.64 | |
| *Ischyromys typus* [2] | 6.93 | | 1109.01 | | 0.53 | 0.70 | | 0.77 | |
| *Paramys copei* [6] | 7.17 | | 1029.89 | | 0.57 | 0.76 | | 0.83 | |
| *Paramys delicatus* [6] | 11.97 | | 2704.83 | | 0.50 | 0.62 | | 0.75 | |
| *Coendou spinosus* [This study] | 13.33 | | 1040 | | 1.06 | 1.41 | | 1.55 | |
| *Erethizon dorsatum* [5] | 27.9 | | 5160 | | 0.76 | 0.90 | | 1.16 | |
| *Erethizon dorsatum* [5] | 28.7 | | 3430 | | 1.02 | 1.26 | | 1.55 | |
| *Erethizon dorsatum* [5] | 25.1 | | 4980 | | 0.70 | 0.83 | | 1.07 | |
| *Erethizon dorsatum* [5] | 27.2 | | 4980 | | 0.76 | 0.90 | | 1.16 | |
| *Erethizon dorsatum* [5] | 27.1 | | 4640 | | 0.79 | 0.95 | | 1.20 | |
| *Erethizon dorsatum* [5] | 27.1 | | 6200 | | 0.65 | 0.77 | | 1.00 | |
| *Erethizon dorsatum* [33] | 24 | | 6640 | | 0.55 | 0.64 | | 0.85 | |
| *Erethizon dorsatum* [33] | 30.77 | | 2800 | | 1.26 | 1.56 | | 1.89 | |
| *Erethizon dorsatum* [33] | 21.22 | | 2725 | | 0.88 | 1.10 | | 1.33 | |
| *Erethizon dorsatum* [33] | 19.15 | | 3410 | | 0.69 | 0.84 | | 1.04 | |
| *Erethizon dorsatum* [34] | 24 | | 5000 | | 0.66 | 0.79 | | 1.02 | |
| *Myocastor coypus* [This study] | 13.78 | | 6400 | | 0.32 | 0.38 | | 0.50 | |
| *Myocastor coypus* [35] | 23 | | 3300 | | 0.84 | 1.04 | | 1.27 | |
| *Myocastor coypus* [35] | 19 | | 3380 | | 0.68 | 0.84 | | 1.03 | |
| *Myocastor coypus* [33] | 23 | | 5000 | | 0.64 | 0.76 | | 0.97 | |
| *Myocastor coypus* [36] | 18.7 | | 5300 | | 0.50 | 0.59 | | 0.76 | |
| *Myocastor coypus* [37] | 21 | | 3300 | | 0.77 | 0.95 | | 1.16 | |
| *Myocastor coypus* [38] | 15.64 | | 7450 | | 0.33 | 0.39 | | 0.51 | |
| *Myocastor coypus* [38] | 15.6 | | 7530 | | 0.33 | 0.38 | | 0.51 | |
| *Myocastor coypus* [38] | 17.55 | | 8475 | | 0.34 | 0.39 | | 0.53 | |
| *Myocastor coypus* [38] | 17.43 | | 5510 | | 0.45 | 0.54 | | 0.69 | |
| Table S2. Continued | | | | | | | | |  |
| **Species** | | **Brain mass (g)** | **Body mass (g)** | **EQ Jerison** | | | **EQ Eisemberg** | **EQ Pilleri et al.** | |
| *Myocastor coypus* [38] | | 14.77 | 3800 | 0.49 | | | 0.60 | 0.75 | |
| *Myocastor coypu s* [38] | | 18.35 | 5700 | 0.47 | | | 0.55 | 0.71 | |
| *Myocastor coypus f domestica* [34] | | 18.4 | 3500 | 0.65 | | | 0.79 | 0.98 | |
| *Myocastor coypus* [39] | | 23 | 5000 | 0.64 | | | 0.76 | 0.97 | |
| *Myocastor coypus* [34] | | 9.9 | 780 | 0.95 | | | 1.30 | 1.38 | |
| *Phyllomys dastrix* [this study] | | 2.55 | 339 | 0.43 | | | 0.62 | 0.61 | |
| *Capromys pilorides* [34] | | 11 | 7000 | 0.24 | | | 0.28 | 0.38 | |
| *Dasyprocta agouti* [35] | | 21.6 | 3600 | 0.75 | | | 0.91 | 1.13 | |
| *Dasyprocta agouti* [40] | | 20 | 2684 | 0.84 | | | 1.05 | 1.26 | |
| *Dasyprocta agouti* [38] | | 18.4 | 2390 | 0.84 | | | 1.05 | 1.25 | |
| *Dasyprocta agouti* [38] | | 17 | 2004 | 0.87 | | | 1.11 | 1.29 | |
| *Dasyprocta agouti* [38] | | 18.5 | 2880 | 0.74 | | | 0.92 | 1.12 | |
| *Dasyprocta agouti* [38] | | 18 | 2350 | 0.83 | | | 1.04 | 1.24 | |
| *Dasyprocta agouti* [38] | | 17 | 2550 | 0.74 | | | 0.93 | 1.11 | |
| *Dasyprocta agouti* [38] | | 18.3 | 3172 | 0.69 | | | 0.85 | 1.04 | |
| *Dasyprocta agouti* [38] | | 19.8 | 2371 | 0.90 | | | 1.14 | 1.35 | |
| *Dasyprocta agouti* [34] | | 27 | 2400 | 1.22 | | | 1.54 | 1.83 | |
| *Dasyprocta agouti* [39] | | 19.8 | 2370 | 0.90 | | | 1.14 | 1.35 | |
| *Dasyprocta punctata* [33] | | 18.34 | 3172 | 0.69 | | | 0.85 | 1.04 | |
| *Dasyprocta mexicanus*[40] | | 17.8 | 1527 | 1.09 | | | 1.42 | 1.61 | |
| *Dasyprocta mexicanus* [40] | | 20 | 2300 | 0.93 | | | 1.18 | 1.39 | |
| *Dasyprocta sp* [this study] | | 22.84 | 3700 | 0.77 | | | 0.95 | 1.17 | |
| *Neoreomys australis* [this study] | | 12.58 | 7200 | 0.27 | | | 0.32 | 0.42 | |
| *Dolichotis patagonum* [35] | | 25.66 | 5650 | 0.65 | | | 0.78 | 1.00 | |
| *Dolichotis patagonum* [38] | | 33.5 | 7880 | 0.68 | | | 0.79 | 1.06 | |
| *Dolichotis patagonum* [38] | | 32 | 7500 | 0.68 | | | 0.79 | 1.04 | |
| *Dolichotis patagonum* [38] | | 26 | 5500 | 0.68 | | | 0.80 | 1.04 | |

| Table S2. Continued | | | | | | | | | | | | | | | |  |  |
| --- | --- | --- | --- | --- | --- | --- | --- | --- | --- | --- | --- | --- | --- | --- | --- | --- | --- |
| **Species** | **Brain mass (g)** | | **Body mass (g)** | | | | | | **EQ Jerison** | | **EQ Eisemberg** | | | | **EQ Pilleri et al.** | | |
| *Dolichotis patagonum* [38] | 30 | | 7200 | | | | | | 0.65 | | 0.76 | | | | 1.01 | | |
| *Dolichotis patagonum* [34] | 15 | | 5500 | | | | | | 0.39 | | 0.46 | | | | 0.60 | | |
| *Dolichotis patagonum* [39] | 25.7 | | 5650 | | | | | | 0.66 | | 0.78 | | | | 1.01 | | |
| *Cuniculus paca* [41] | 35.8 | | 5635 | | | | | | 0.92 | | 1.09 | | | | 1.40 | | |
| *Cuniculus paca* [33] | 21.85 | | 3627 | | | | | | 0.75 | | 0.92 | | | | 1.14 | | |
| *Cuniculus paca* [33] | 48 | | 4559 | | | | | | 1.41 | | 1.70 | | | | 2.16 | | |
| *Cuniculus paca* [38] | 33.5 | | 6125 | | | | | | 0.81 | | 0.95 | | | | 1.25 | | |
| *Cuniculus paca* [38] | 33.2 | | 3665 | | | | | | 1.13 | | 1.38 | | | | 1.72 | | |
| *Cuniculus paca* [38] | 37.3 | | 9000 | | | | | | 0.70 | | 0.80 | | | | 1.08 | | |
| *Cuniculus paca* [39] | 26.1 | | 5000 | | | | | | 0.72 | | 0.86 | | | | 1.11 | | |
| *Cavia porcellus* [5] | 5.4 | | 520 | | | | | | 0.68 | | 0.95 | | | | 0.98 | | |
| *Cavia porcellus*[5] | 5.8 | | 650 | | | | | | 0.63 | | 0.87 | | | | 0.91 | | |
| *Cavia porcellus* [42] | 6.4 | | 348 | | | | | | 1.06 | | 1.52 | | | | 1.50 | | |
| *Cavia porcellus* [42] | 4.4 | | 648 | | | | | | 0.48 | | 0.66 | | | | 0.69 | | |
| *Cavia porcellus* [42] | 3.8 | | 493 | | | | | | 0.50 | | 0.70 | | | | 0.71 | | |
| *Cavia porcellus* [33] | 4.1 | | 361 | | | | | | 0.66 | | 0.95 | | | | 0.94 | | |
| *Cavia porcellus* [33] | 3.8 | | 324 | | | | | | 0.66 | | 0.95 | | | | 0.93 | | |
| *Cavia porcellus* [33] | 3.32 | | 214.94 | | | | | | 0.76 | | 1.13 | | | | 1.06 | | |
| *Cavia porcellus* [33] | 2.28 | | 214.57 | | | | | | 0.52 | | 0.78 | | | | 0.73 | | |
| *Cavia porcellus* [33] | 4 | | 432 | | | | | | 0.57 | | 0.81 | | | | 0.82 | | |
| *Cavia porcellus* [33] | 4.23 | | 456 | | | | | | 0.58 | | 0.82 | | | | 0.83 | | |
| *Cavia porcellus* [40] | 4.73 | | 700 | | | | | | 0.49 | | 0.67 | | | | 0.71 | | |
| *Cavia porcellus* [40] | 4.54 | | 675 | | | | | | 0.48 | | 0.66 | | | | 0.70 | | |
| *Cavia porcellus* [32] | 4.28 | | 971 | | | | | | 0.36 | | 0.48 | | | | 0.52 | | |
| *Cavia porcellus* [13] | 4.94 | | 900 | | | | | | 0.43 | | 0.58 | | | | 0.63 | | |
| *Cavia porcellus* [40] | 5 | | 500 | | | | | | 0.65 | | 0.91 | | | | 0.93 | | |
| *Cavia porcellus* [Present study] | 4.32 | | 1000 | | | | | | 0.35 | | 0.47 | | | | 0.51 | | |
| *Cavia aperea f. porcellus* [34] | 4.57 | | 485 | | | | | | 0.60 | | 0.85 | | | | 0.87 | | |
| *Cavia aperea* [38] | 2.7 | | 163 | | | | | | 0.74 | | 1.13 | | | | 1.03 | | |
| *Cavia aperea* [38] | 3 | | 260 | | | | | | 0.60 | | 0.89 | | | | 0.85 | | |
| Table S2. Continued | | | | | | | | | | | | | | | |  |  |
| **Species** | **Brain mass (g)** | | | **Body mass (g)** | | | **EQ Jerison** | | | | **EQ Eisemberg** | | | **EQ Pilleri et al.** | | | |
| *Cavia aperea* [38] | 3.9 | | | 430 | | | 0.56 | | | | 0.79 | | | 0.80 | | | |
| *Cavia aperea* [38] | 5.46 | | | 647 | | | 0.60 | | | | 0.82 | | | 0.86 | | | |
| *Cavia aperea* [38] | 4.2 | | | 460 | | | 0.58 | | | | 0.81 | | | 0.82 | | | |
| *Cavia aperea* [38] | 4.7 | | | 792 | | | 0.45 | | | | 0.61 | | | 0.65 | | | |
| *Cavia aperea* [38] | 4.1 | | | 540 | | | 0.50 | | | | 0.70 | | | 0.72 | | | |
| *Galea spixii* [42] | 6.2 | | | 672 | | | 0.66 | | | | 0.91 | | | 0.95 | | | |
| *Hydrochaerus hydrochaeris* [5] | 85 | | | 31000 | | | 0.69 | | | | 0.73 | | | 1.12 | | | |
| *Hydrochaerus hydrochaeris* [5] | 61.4 | | | 24130 | | | 0.59 | | | | 0.63 | | | 0.95 | | | |
| *Hydrochaerus hydrochaeris* [33] | 76 | | | 29500 | | | 0.64 | | | | 0.68 | | | 1.03 | | | |
| *Hydrochaerus hydrochaeris* [13] | 55 | | | 10000 | | | 0.96 | | | | 1.09 | | | 1.49 | | | |
| *Hydrochaerus hydrochaeris* [13] | 53 | | | 12000 | | | 0.82 | | | | 0.92 | | | 1.28 | | | |
| *Hydrochaerus hydrochaeris* [13] | 53 | | | 13000 | | | 0.77 | | | | 0.87 | | | 1.22 | | | |
| *Hydrochaerus hydrochaeris* [13] | 61 | | | 17000 | | | 0.74 | | | | 0.82 | | | 1.18 | | | |
| *Hydrochaerus hydrochaeris* [13] | 52 | | | 28000 | | | 0.45 | | | | 0.48 | | | 0.73 | | | |
| *Hydrochaerus hydrochaeris* [37] | 61.4 | | | 24031 | | | 0.59 | | | | 0.64 | | | 0.95 | | | |
| *Hydrochaerus hydrochaeris* [40] | 75 | | | 28500 | | | 0.65 | | | | 0.69 | | | 1.04 | | | |
| *Hydrochaerus hydrochaeris* [33] | 52.21 | | | 27670 | | | 0.46 | | | | 0.49 | | | 0.74 | | | |
| *Hydrochaerus hydrochaeris* [38] | 75 | | | 26700 | | | 0.68 | | | | 0.72 | | | 1.08 | | | |
| *Hydrochaerus hydrochaeris* [38] | 73 | | | 34300 | | | 0.56 | | | | 0.58 | | | 0.90 | | | |
| *Hydrochaerus hydrochaeris* [38] | 75.5 | | | 33000 | | | 0.59 | | | | 0.62 | | | 0.95 | | | |
| *Hydrochaerus hydrochaeris* [38] | 82 | | | 54000 | | | 0.46 | | | | 0.47 | | | 0.75 | | | |
| *Hydrochaerus hydrochaeris* [38] | 76.02 | | | 29500 | | | 0.64 | | | | 0.68 | | | 1.03 | | | |
| *Hydrochaerus hydrochaeris* [38] | 75 | | | 28000 | | | 0.66 | | | | 0.69 | | | 1.05 | | | |
| *Hydrochaerus hydrochaeris* [42] | 71 | | | 32500 | | | 0.56 | | | | 0.59 | | | 0.90 | | | |
| *Hydrochaerus hydrochaeris* [Present study] | 90.24 | | | 55500 | | | 0.50 | | | | 0.50 | | | 0.82 | | | |
| *Dolicavia minuscula* [19] | 5.72 | | | 534.52 | | | 0.71 | | | | 0.99 | | | 1.02 | | | |
| *Chinchilla brevicaudata* [35] | 7.8 | | | 520 | | | 0.98 | | | | 1.38 | | | 1.41 | | | |
|  |  | | |  | | |  | | | |  | | |  | | | |
| Table S2. Continued | | | | | | | | | | | | | | | |  |  |
| **Species** | **Brain mass (g)** | | | | **Body mass (g)** | | | **EQ Jerison** | | | **EQ Eisemberg** | | **EQ Pilleri et al.** | | | |  |
| *Chinchilla brevicaudata* [5] | 6.9 | | | | 320 | | | 1.21 | | | 1.75 | | 1.71 | | | |  |
| *Chinchilla brevicaudata* [5] | 6.4 | | | | 425 | | | 0.92 | | | 1.31 | | 1.32 | | | |  |
| *Chinchilla brevicaudata* [5] | 8.9 | | | | 470 | | | 1.20 | | | 1.70 | | 1.72 | | | |  |
| *Chinchilla brevicaudata* [5] | 6 | | | | 450 | | | 0.83 | | | 1.18 | | 1.19 | | | |  |
| *Chinchilla laginera* [33] | 5.2 | | | | 432 | | | 0.74 | | | 1.05 | | 1.06 | | | |  |
| *Chinchilla laginera* [38] | 5.2 | | | | 380 | | | 0.81 | | | 1.16 | | 1.15 | | | |  |
| *Chinchilla laginera* [38] | 5.05 | | | | 385 | | | 0.78 | | | 1.12 | | 1.11 | | | |  |
| *Chinchilla laginera* [38] | 5.1 | | | | 460 | | | 0.70 | | | 0.99 | | 1.00 | | | |  |
| *Chinchilla laginera* [38] | 4.9 | | | | 385 | | | 0.76 | | | 1.08 | | 1.08 | | | |  |
| *Chinchilla laginera* [38] | 5.32 | | | | 370 | | | 0.84 | | | 1.21 | | 1.20 | | | |  |
| *Chinchilla laginera* [38] | 5.5 | | | | 385 | | | 0.85 | | | 1.21 | | 1.21 | | | |  |
| *Chinchilla laginera* [39] | 6.4 | | | | 425 | | | 0.92 | | | 1.31 | | 1.32 | | | |  |
| *Chinchilla lanigera* [Present study] | 5.4 | | | | 430 | | | 0.77 | | | 1.10 | | 1.10 | | | |  |
| *Chinchilla velliger F. Domestica* [34] | 6 | | | | 500 | | | 0.78 | | | 1.09 | | 1.11 | | | |  |
| *Lagostomus maximus* [Present study] | 16.96 | | | | 6000 | | | 0.42 | | | 0.49 | | 0.64 | | | |  |
| *Lagostomus maximus* [38] | 20 | | | | 6630 | | | 0.46 | | | 0.54 | | 0.71 | | | |  |
| *Lagostomus maximus* [38] | 21 | | | | 6385 | | | 0.49 | | | 0.58 | | 0.76 | | | |  |
| *Lagostomus maximus* | 17.2 | | | | 5965 | | | 0.42 | | | 0.50 | | 0.65 | | | |  |
| *Lagostomus maximus* [38] | 18 | | | | 3930 | | | 0.59 | | | 0.71 | | 0.89 | | | |  |
| *Lagostomus maximus* [38] | 16.5 | | | | 3765 | | | 0.55 | | | 0.67 | | 0.84 | | | |  |
| *Lagostomus maximus* [38] | 16.3 | | | | 2940 | | | 0.64 | | | 0.80 | | 0.97 | | | |  |
| *Lagostomus maximus* [34] | 17 | | | | 7000 | | | 0.38 | | | 0.44 | | 0.58 | | | |  |
| *Lagidium maximus* [40] | 8.8 | | | | 3854 | | | 0.29 | | | 0.35 | | 0.44 | | | |  |
| *Lagidium peruanum* [38] | 12.2 | | | | 1575 | | | 0.73 | | | 0.95 | | 1.08 | | | |  |
| *Lagidium peruanum* [38] | 14.5 | | | | 1990 | | | 0.74 | | | 0.95 | | 1.11 | | | |  |
| *Lagidium peruanum* [38] | 12 | | | | 1395 | | | 0.78 | | | 1.02 | | 1.15 | | | |  |
| *Lagidium peruanum* [38] | 11.1 | | | | 1500 | | | 0.69 | | | 0.90 | | 1.02 | | | |  |
| *Lagidium peruanum* [38] | 11.8 | | | | 1265 | | | 0.82 | | | 1.08 | | 1.21 | | | |  |
| *Lagidium peruanum* [38] | 13.2 | | | | 1125 | | | 0.99 | | | 1.32 | | 1.46 | | | |  |
| *Lagidium peruanum* [38] | 11.7 | | | | 1095 | | | 0.90 | | | 1.19 | | 1.31 | | | |  |
|  |  |  | | | |  | | | |  | |  | | | |  |  |
| Table S2. Continued |  |  | | | |  | | | |  | |  | | | |  |  |
| **Species** | **Brain mass (g)** | **Body mass (g)** | | | | **EQ Jerison** | | | | **EQ Eisemberg** | | **EQ Pilleri et al.** | | | |  |  |
| *Lagidium peruanum* [38] | 13 | 1440 | | | | 0.83 | | | | 1.08 | | 1.22 | | | |  |  |
| *Lagidium peruanum* [38] | 12.7 | 1225 | | | | 0.90 | | | | 1.19 | | 1.33 | | | |  |  |
| *Lagidium peruanum* [38] | 12.6 | 1510 | | | | 0.78 | | | | 1.01 | | 1.15 | | | |  |  |
| *Lagidium peruanum* [38] | 11.9 | 1335 | | | | 0.80 | | | | 1.05 | | 1.18 | | | |  |  |
| *Lagidium peruanum* [38] | 11.9 | 1325 | | | | 0.80 | | | | 1.05 | | 1.18 | | | |  |  |
| *Lagidium peruanum* [38] | 12.3 | 1115 | | | | 0.93 | | | | 1.24 | | 1.37 | | | |  |  |
| *Lagidium peruanum* [38] | 11 | 1075 | | | | 0.85 | | | | 1.14 | | 1.25 | | | |  |  |
| *Lagidium peruanum* [38] | 11.2 | 1030 | | | | 0.89 | | | | 1.19 | | 1.31 | | | |  |  |
| *Lagidium peruanum* [38] | 11.2 | 1125 | | | | 0.84 | | | | 1.12 | | 1.24 | | | |  |  |
| *Lagidium peruanum* [38] | 11.7 | 1230 | | | | 0.83 | | | | 1.09 | | 1.22 | | | |  |  |
| *Lagidium viscacia* [39] | 12.4 | 2460 | | | | 0.55 | | | | 0.69 | | 0.83 | | | |  |  |
| *Lagidium viscacia* [41] | 16 | 2252 | | | | 0.76 | | | | 0.96 | | 1.13 | | | |  |  |
| *Lagidium viscacia* [41] | 15.2 | 2350 | | | | 0.70 | | | | 0.88 | | 1.05 | | | |  |  |
| *Lagidium viscacia* [41] | 14.8 | 3845 | | | | 0.49 | | | | 0.60 | | 0.74 | | | |  |  |
| *Dinomys branickii* [Present study] | 41.43 | 15000 | | | | 0.55 | | | | 0.61 | | 0.87 | | | |  |  |
| *Neoepiblema acreensis* [Present study] | 47.23 | 79500 | | | | 0.21 | | | | 0.20 | | 0.34 | | | |  |  |
| *Hystrix cristada* [35] | 36.5 | 7036.5 | | | | 0.80 | | | | 0.94 | | 1.24 | | | |  |  |
| *Hystrix cristada* [34] | 32 | 15000 | | | | 0.42 | | | | 0.47 | | 0.67 | | | |  |  |
| *Hystrix cristada* [39] | 37 | 10000 | | | | 0.64 | | | | 0.73 | | 1.00 | | | |  |  |

**Table S3**. Encephalization quotient (EQ) estimates of the brain endocasts studied here. The body mass employed in the equations is the average of the eight results present in the Table S3. The body mass of other rodents is based on the literature.

| **Species** | **Total Endocast Volume(mm³)** | **Brain Mass (g)** | **Body Mass (g)** | **Jerison (1973)** | **Eisenberg (1981)** | **Pilleri et al. (1984)** |
| --- | --- | --- | --- | --- | --- | --- |
| *Neoepiblema acreensis* | 49682.06 | 47.31 | 79750 | 0.20 | 0.20 | 0.33 |
| *Neoreomys australis* | 13208.44 | 12.58 | 5120 | 0.34 | 0.41 | 0.52 |
| *Chinchilla lanigera* | 5669.75 | 5.40 | 612[25] | 0.61 | 0.85 | 0.88 |
| *Dinomys branickii* | 43502.22 | 41.43 | 15000[26] | 0.55 | 0.61 | 0.87 |
| *Lagostomus maximus* | 17803.59 | 16.96 | 6150[27] | 0.41 | 0.48 | 0.63 |
| *Hydrochaerus hydrochaeris* | 94746.94 | 90.24 | 69950[28] | 0.43 | 0.42 | 0.70 |
| *Cavia porcelus* | 4532.05 | 4.32 | 637[4] | 0.48 | 0.66 | 0.69 |
| *Dasyprocta* sp. | 23980.98 | 22.84 | 3700[29] | 0.77 | 0.95 | 1.17 |
| *Coendou spinosus* | 13831.53 | 13.17 | 750[30] | 1.32 | 1.80 | 1.91 |
| *Myocastor coypus* | 16432.1 | 15.65 | 6530[31] | 0.36 | 0.43 | 0.56 |
| *Phyllomys dasythrix* | 2573.77 | 2.45 | 200[32] | 0.61 | 0.92 | 0.85 |

**Table S4**. Encephalization Quotients of *N. acreensis* (UFAC 4515) with increasing in the percentage (10 – 40%) of the endocranial volume to simulate the loss of the original volume due to the taphonomic modifications.

| *N. acreensis* | Total Endocast Volume(mm³) | Brain Mass (g) | Body Mass (g) | Jerison (1973) | Eisenberg (1987) | Pilleri et al. (1984) |
| --- | --- | --- | --- | --- | --- | --- |
|  | 49682,06 | 47,31 | 79750 | 0,20 | 0,20 | 0,33 |
| 10% | 54650,27 | 52,04 | 79750 | 0,23 | 0,23 | 0,37 |
| 20% | 59618,47 | 56,78 | 79750 | 0,25 | 0,24 | 0,41 |
| 30% | 64586,68 | 61,51 | 79750 | 0,27 | 0,26 | 0,44 |
| 40% | 69554,88 | 66,24 | 79750 | 0,29 | 0,28 | 0,47 |

**Table S5**. Body estimates of *N. acreensis* based on the equations of Millien [7].

| **Character** | **Equation** | **Measurement (mm)** | **Body mass (kg)** |
| --- | --- | --- | --- |
| TL | LogM = -6,5512+3,4957 logX | 276.3 | 96.19 |
| FW | LogM = -4,0638+2,9747 logX | 71.55 | 28.39 |
| ZL | LogM = -5,0698 + 3,3028 logX | 130.00 | 81.68 |
| DL | LogM = -4,7479+3,6052 logX | 71.26 | 85.50 |
| RL | LogM = -5,0979 + 3,1616 logX | 167.5 | 85,81 |
| BCL | LogM = -4,9631 + 3,847 logX | 64.10 | 97,25 |
| UTRW | LogM=-3,0383+2,6979 logX | 70.05 | 87,18 |
| Skull | LogM= -6.3319+3.4878 logX | 226.3 | 75,99 |

**Table S6**. Linear measurements (in mm) and volumes of the brain endocasts of the caviomorphs analyzed herein.

| **Measurements** | ***Neoepiblema acreensis*** | ***Chinchilla lanigera*** | ***Dinomys branickii*** | ***Lagostomus maximus*** | ***Hydrochaerus hydrochaeris*** | ***Cavia porcellus*** | ***Dasyprocta* sp** | ***Coendou spinosus*** | ***Myocastor coypus*** | ***Phylomys dasythrix*** | ***Neoreomys australis*** |
| --- | --- | --- | --- | --- | --- | --- | --- | --- | --- | --- | --- |
| Total endocast length | 82.88 | 38.84 | 70.73 | 56.23 | 93.44 | 37.58 | 61.55 | 44.01 | 50.38 | 30.40 | 54.65 |
| Olfactory bulbs length | 11.15 | 5.05 | 9.14 | 7.36 | 11.26 | 5.64 | 10.64 | 6.41 | 6.67 | 4.19 | 10.20 |
| Olfactory bulbs width | 13.34 | 7.44 | 15.74 | 9.27 | 25.56 | 7.91 | 13.05 | 14.31 | 12.53 | 6.22 | 9.18 |
| Olfactory bulbs height | 6.34 | 5.14 | 7.58 | 6.46 | 16.48 | 5.56 | 8.77 | 8.51 | 7.68 | 4.46 | 8.64 |
| Maximum cerebrum length | 54.43 | 22.85 | 45.96 | 34.76 | 58.46 | 23.03 | 38.46 | 28.33 | 31.90 | 17.40 | 30.49 |
| Maximum cerebrum width | 55.87 | 25.00 | 45.01 | 39.00 | 62.95 | 23.48 | 37.21 | 30.34 | 31.05 | 16.97 | 30.86 |
| Maximum cerebrum height | 24.09 | 14.67 | 30.51 | 19.98 | 30.68 | 12.79 | 24.11 | 20.69 | 19.65 | 10.93 | 20.37 |
| Cerebellum width (without paraflocculi) | 32.17 | 12.61 | 30.65 | 20.32 | 30.32 | 7.30 | 21.72 | 24.04 | 20.53 | 12.47 | 19.81 |
| Maximum cerebellum length | 13.97 | 9.81 | 11.64 | 7.55 | 13.46 | 12.04 | 11.74 | 9.04 | 9.26 | 5.91 | 15.05 |
| Ratio linear maasurements (%) |  |  |  |  |  |  |  |  |  |  |  |
| OL/TL | 13.45 | 13.00 | 12.92 | 13.09 | 12.05 | 15.00 | 17.28 | 14.56 | 13.24 | 13.78 | 18.66 |
| CRML/TL | 65.67 | 58.83 | 64.98 | 61.82 | 62.56 | 61.28 | 62.49 | 64.37 | 63.32 | 57.24 | 55.79 |
| CLML/TL | 16.86 | 25.26 | 16.43 | 13.42 | 14.40 | 32.03 | 19.07 | 20.54 | 18.38 | 19.44 | 0.28 |
| CLW/CRMW | 57.58 | 50.44 | 68.10 | 52.10 | 48.16 | 31.09 | 58.37 | 79.23 | 66,12 | 73.48 | 49.36 |
| OW/CRMW | 23.87 | 29.76 | 34.97 | 23.77 | 40.60 | 33.69 | 35.07 | 47.17 | 40.35 | 36.65 | 29.74 |
| Olfactory bulbs volume | - | 112.40 | 1073.78 | 267.24 | 2278.62 | 124.45 | 865.35 | 308.58 | 312.43 | 75.52 | 436.48 |

**Table S7.** Body estimates of *Neoreomys australis* based on the equations of Millien [7].

| **Character** | **Equation** | **Measurement (mm)** | **Body mass (kg)** |
| --- | --- | --- | --- |
| TL | LogM = -6,5512+3,4957 logX | 119.52 | 5.14 |
| DL | LogM = -4,7479+3,6052 logX | 29.47 | 3.54 |
| UTRW | LogM=-3,0383+2,6979 logX | 27.04 | 6.69 |

**References**

1. Zurita-Altamirano D, Buffetaut E, Forasiepi AM, Kramarz A, Carrillo JD, Aguirre-Fernández G, Carlini AA, Scheyer TM, Sánchez-Villagra MR. 2019 The Allemann collection from the Santa Cruz Formation (late early Miocene), Argentina, in Zurich, Switzerland. *Swiss J. Palaeontol.* (doi:10.1007/s13358-019-00185-5)

2. Bertrand OC, Silcox MT. 2016 First virtual endocasts of a fossil rodent: *Ischyromys typus* (Ischyromyidae, Oligocene) and brain evolution in rodents. *J. Vertebr. Paleontol.* **36**, e1095762. (doi:10.1080/02724634.2016.1095762)

3. Jerison H. 1973 *Evolution of the brain and intelligence*. New York: Academic Press.

4. Eisenberg J. 1981 *The mammalian radiations: an analysis of trends in evolution, adaptation, and behavior*. Chicago: University of Chicago Press.

5. Pilleri G, Gihr M, Kraus C. 1984 Cephalization in rodents with particular reference to the canadian beaver (*Castor canadensisi*). In *Investigations on Behavers* (ed G Pilleri), pp. 11–102. Berne: Brain Anatomy Institute.

6. Bertrand OC, Amador-Mughal F, Silcox MT. 2016 Virtual endocasts of Eocene *Paramys* ( Paramyinae ): oldest endocranial record for Rodentia and early brain evolution in Euarchontoglires. *Proc. R. Soc. B* **283**, 1–8. (doi:http://dx.doi.org/10.1098/rspb.2015.2316)

7. Millien V. 2008 The largest among the smallest: the body mass of the giant rodent *Josephoartigasia monesi*. *Proc. R. Soc. B Biol. Sci.* **275**, 1953–1955. (doi:10.1098/rspb.2008.0087)

8. Herculano-Houzel S, Ribeiro P, Campos L, Valotta da Silva A, Torres LB, Catania KC, Kaas JH. 2011 Updated neuronal scaling rules for the brains of Glires (Rodents/Lagomorphs). *Brain. Behav. Evol.* **78**, 302–314. (doi:10.1159/000330825)

9. Maddison W, Maddison D. 2015 Mesquite: a modular system for evolutionary analysis.

10. Upham N, Patterson B. 2015 Evolution of the caviomorph rodents: a complete phylogeny and time tree of living genera. In *Biology of caviomorph rodents: diversity and evolution* (eds A Vassallo, D Antonucci), pp. 63–120. Buenos Aires: Sociedad Argentina para el estudio de los Mamíferos.

11. Pérez ME, Vallejo-Pareja MC, Carrillo JD, Jaramillo C. 2017 A New Pliocene capybara (Rodentia, Caviidae) from Northern South America (Guajira, Colombia), and its implications for the great american biotic interchange. *J. Mamm. Evol.* **24**, 111–125. (doi:10.1007/s10914-016-9356-7)

12. Kerber L, Ferreira JD, Negri FR. 2019 A reassessment of the cranial morphology of *Neoepiblema acreensis* (Rodentia: Chinchilloidea), a Miocene rodent from South America. *J. Morphol.* , jmor.21067. (doi:10.1002/jmor.21067)

13. Campos G., Welker W. 1976 1976 Comparisons between brains of a large and a small hystricomorph rodent: capybara. *Hydrochoerus* and guinea pig. *Cavia* neocortical projection regions and measurements of brain subdivisions. *Brain Behav. Evol.* **13**, 243–266.

14. Wahlert J. 1985 Cranial foramina of rodents. In *Evolutionary relationships among rodents: a multidisciplinary analysis* (eds WP Luckett, J. Hartenberger), pp. 311–332. New York: Plenum Press.

15. Novacek M. 1982 The brain of *Leptictis dakotensis*. an Oligocene leptictid. Eutheria. Mammalia. from North America. *J. Paleontol.* **56**, 1177–1186.

16. Nasif N, Abdala F. 2015 Craniodental ontogeny of the pacarana *Dinomys branickii* Peters 1873 ( Rodentia, Hystricognathi, Caviomorpha, Dinomyidae). **96**, 1224–1244. (doi:10.1093/jmammal/gyv131)

17. Wahlert J. 1974 The cranial foramina of protogomorphous rodents: an anatomical and phylogenetic study. *Bull. Museum Comp. Zool.* **146**, 263–410.

18. Dozo MT, Martínez G. 2016 First digital cranial endocasts of late Oligocene Notohippidae (Notoungulata): implications for endemic South American ungulates brain evolution. *J. Mamm. Evol.* **23**, 1–16. (doi:10.1007/s10914-015-9298-5)

19. Dozo MT, Vucetich MG, Candela AM. 2004 Skull anatomy and neuromorphology of *Hypsosteiromys* , a Colhuehuapian erethizontid rodent from Argentina. *J. Vertebr. Paleontol.* **24**, 228–234. (doi:10.1671/18.1)

20. Dozo M. 1997 Paleoneurología de *Dolicavia minuscula* (Rodentia, Caviidae) y *Paedotherium insigne* (Notoungulata, Hegetotheriidae) del Plioceno de Buenos Aires, Argentina. *Ameghiniana* **34**, 427–435.

21. Madozzo-Jaén MC. 2019 Systematic and phylogeny of *Prodolichotis prisca* (Caviidae, Dolichotinae) from the Northwest of Argentina (late Miocene–early Pliocene): advances in the knowledge of the evolutionary history of maras. *Comptes Rendus Palevol* **18**, 33–50. (doi:10.1016/j.crpv.2018.07.003)

22. Dozo M.1997 Primer estudio paleoneurológico de un roedor caviomorfo (Cephalomyidae) y sus posibles implicancias filogenéticas. *Mastozoología Neotrop.* **4**, 89 – 96.

23. Rasia L, Candela A. 2019 Upper molar morphology, homologies and evolutionary patterns of chinchilloid rodents (Mammalia, Caviomorpha). *J. Anat.* **234**, 50–65. (doi:10.1111/joa.12895)

24. Kerber L, Negri FR, Ferigolo J, Mayer EL, Ribeiro AM. 2017 Modifications on fossils of neoepiblemids and other South American rodents. *Lethaia* **50**, 149–161. (doi:10.1111/let.12183)

25. Spotorno A, Zuleta C, Valladares J, Deane A, Jiménez J. 2004 *Chinchilla laniger*. *Mamm. species* **758**, 1–9.

26. Patton J. 2015 Genus *Dinomys*. In *Mammals of South America* (eds J. Patton, G. Pardiñas, UF. D’Elía), pp. 784–786. Chicago: University of Chicago Press.

27. Jackson J, Branch L, Vilarreal D. 1996 *Lagostomus maximus*. *Mamm. Species* **543**, 1–6.

28. Mones A, Ojasti J. 1986  *Hydrochoerus hydrochaeris*. *Mamm. Species* **246**, 1–7.

29. Emmons L, Feer F. 1997 *Neotropical rainforest mammals: a Field Guide*. 2^a^. Chicago: University of Chicago Press.

30. Roach N, Naylor L. 2016 *Coendou spinosus. IUCN Red List of Threatened Species. IUCN*.

31. Woods C, Contreras L, Willner-Chapman G, Whidden H. 1992 *Myocastor coypus*. *Mamm. Species* **98**, 1–8.

32. Leite Y. 2003 Evolution and systematics of the atlantic tree rats. Genus *Phyllomys* (Rodentia. Echimyidae). With description of two new species. *Univ. Calif. Publ. Zool.* **132**, 1–137.

33. Sacher G., Staffeldt E. 1974 Relation of gestation time to brain weight for placental mammals: implications for the theory of vertebrate growth. *Am. Nat.* **108**, 593–615.

34. Brauer K, Schober W. 1970 *Katalog der säugetiergehirn. Catalogue of mammalian brains.* Jena, Germany: Gustav Fischer Verlag.

35. Pilleri G. 1959 Beiträge zur vergleichenden morphologie des nagetiergehirnes. L. Beitrag: Sciuromorpha. *Acta Anat. Suppl.* **38**, 1–42.

36. Compoint-Monmignaut C. 1973 L’encéphalisation chez les rongeurs. *Comptes rendus Hebd. des Séances l’Académie des Sci. Paris* **277**, 861–863.

37. Nikitenko MF. 1966 On the brain structure of the beaver in relation to peculiarities of its mode of life and evolution. *Zool. Zhurnal Moskva* **45**, 261–274.

38. Weidemann W. 1967 Vergleichende untersuchungen an gehirnen südamerikanischer nagetiere. *Zeitschrift für Wissenschaftliche Zool.* **181**, 67–187.

39. Mangold-Wirz K. 1966 Cerebralisation und ontogenesemodus bei eutherien. *Cells Tissues Organs* **63**, 449–508.

40. Brummelkamp R. 1939 Das Wachstum der gehirnmasse mit kleinen cephalisierungsspriingen (sog. V2-sprüngen) bei den rodentiern. *Acta Neerl. Morphol. und Pathol.* **2**, 188–194.

41. Warncke P. 1908 Mitteilungen neuer gehirn- und körpergewichtsbestimmungen bei säugern, nebst zusammmenstellung der gesamten bisher beobachteten absoluten und relativen gehirngewichte bei den verschiedenen species. *J. für Psychol. und Neurol.* **13**, 355–403.

42. Anthony R. 1928 *Leçons sur le Cerveau: Encéphale envisagé dans son Ensemble. Télencéphale.* Doin, Paris, France: Télencéphale.
